# Evaluating the Investment Projects of Spinal Medical Device Firms Using the Real Option and DANP-mV Based MCDM Methods

**DOI:** 10.3390/ijerph17093335

**Published:** 2020-05-11

**Authors:** Chi-Yo Huang, Hong-Ling Hsieh, Hueiling Chen

**Affiliations:** 1 Department of Industrial Education, National Taiwan Normal University, Taipei 106, Taiwan; cyhuang66@ntnu.edu.tw (C.-Y.H.); charles19810629@gmail.com (H.-L.H.); 2 Graduate Institute of Management, National Taiwan Normal University, Taipei 106, Taiwan

**Keywords:** real option analysis, venture capitalists (VCs), spinal device, biotechnology, multiple criteria decision making (MCDM)

## Abstract

In an era of global aging, spinal and other joint degeneration issues have become a major problem for many elders. Bone-related operations have become the largest percentage of surgeries, accounting for 40% of the top 10 operations in the United States. Further, these spine-related operations are now ranked second among all bone-related operations. Due to this enormous and daily increasing market demand, more and more firms have started to pay closer attention to related medical devices and products. The global venture capitalists (VCs) have also started to follow the mega trend and will continue to invest heavily in this industry. Although most VCs recognize that investing in firms that produce innovative spinal products or devices is a must, very few practical managers or research scholars have defined the appropriate evaluation methods for these firms to use. The traditional net present value (NPV) method, which does not consider operation flexibility and changes in strategy, is far from the reality. The real option method can reveal the vagueness and flexibilities of the values being embedded in the investment projects at spinal medical device firms. However, the real option method is strictly quantitative. Usually, the evaluation aspects contain qualitative factors or local criteria which are hard to quantify in monetary terms. Thus, the adoption of multiple criteria decision making (MCDM) methods that can manipulate both quantitative and qualitative factors will be very helpful in evaluating and selecting investment cases like the spinal medical device firms, where both quantitative and qualitative factors should be considered. An analytical framework that consists of hybrid MCDM methods and the real option method will thus be very useful to evaluate the newly established firms producing spinal medical devices. Therefore, the authors propose a real option valuation as well as the Decision-Making Trial and Evaluation Laboratory (DEMATEL) based analytic network process (DANP) and the modified VIšekriterijumsko KOmpromisno Rangiranje (VIKOR) method (DANP-mV) based MCDM framework for evaluating the investment projects offered by these firms of spinal medical devices. An empirical study based on three newly established spinal medical device companies specializing in vertebral compression fracture (VCF) surgical devices was used to demonstrate the feasibility of the proposed analytical framework. Sensitivity analysis is performed to determine the influence of modeling parameters on ranking results of alternatives. This analytical framework can thus serve as a tool for VCs to use to determine the value of a potential candidate for investment. The proposed method can also serve as an effective and efficient tool for investment projects in other fields.

## 1. Introduction

In an era of global aging, both the marketing and adoption of interventions asserting to prevent, delay, or reverse aging have increased significantly in recent years [1]. According to the United Nations, the world has entered an era of growing older [2]. The global ratio of our aging population has increased from 8% in the 1950s to 10% as of 2000 and is estimated to reach 61% by 2100 [2]. The increasing speed of the number of people reaching age 60 or older is projected to be three times faster than that of the population growth. Globally, the increasing speed of the number of people attaining age 80 or older, the so-called “oldest-old” people, is also growing [3]. In the past, an aging population occurred mostly in developed countries. Today, that population also appears in developing countries [2]. 

The global aging era has arrived; thus, demands for health care and medical facilitation have also increased tremendously. Global spinal-related diseases are growing steadily due to the growth of the aging and obese population. Therefore, devices for spinal degeneration and other joint degeneration health concerns will play dominant roles in the global market of bone-related medical devices. According to the statistics by Steiner, Karaca, Moore, Imshaug, and Pickens [4], the number of bone-related operations accounts for around 40% of the inpatient invasive, therapeutic surgeries in the United States. Spine surgery is one of the most common surgeries for adults greater than 65 years of age [5]. Further, according to Markets and Markets [6], a leading medical market research firm, the market size of the global spinal implants and surgery devices reached $10.3B USD in of 2019. The market is expected to reach $13.8B USD in 2025, with a compound annual growth rate (CAGR) of 5.0%.

The human spine is made up of 33 vertebrae and can further be divided into cervical (7 vertebras), thoracic (12 vertebras), lumbar (5 vertebras), sacral, and coccygeal elements. According to the different sites of disease occurrences, spinal surgeries can further be divided into five categories: fusion surgery, non-fusion surgery, spinal decompression surgery, vertebral compression fracture (VCF) surgery, and spine biologics surgery. VCF is believed to account for the third-largest market for spinal surgical devices. That global VCF market size is expected to reach $1.109B USD in 2022, with a CAGR of 5.7% from 2014 to 2022 [7]. Therefore, spinal surgical device firms with great growth potential have become key investment targets of venture capitalists (VCs), as they focus on high growth industries. The choice of those target firms with high-growth opportunities is very critical for these VCs. 

The investment valuation of the target firms by VCs is based on expected cash flows for an entire project. VCs also consider other qualitative factors, including the nature of the management team, the related experience of the management team, the market, and the products. Traditionally, VCs use discounted cash flow (DCF) methods, including net present value (NPV) or internal rate of return (IRR), to evaluate an investment project. However, these methods use only quantitative data. Moreover, all these methods assume the same level of risk throughout the entire time horizon of a project. Any flexible market condition after implementing a project is not considered. Another drawback of the DCF methods is that they exclude the value of any different options that may exist in the investment. Because these DCF valuation methods cannot reflect the fast-changing and widely fluctuating market situation, valuations based on traditional methods often lead to incorrect results. In addition, the conflicting results of NPV and IRR may occur because of different cash flow patterns and the size of the investment. Such valuation results can mislead the eventual investment decisions made by VCs. 

The valuation of investment projects by considering strategic and operating options was started in 1977 by Myers [8], whose innovative concept regarding real options was to identify optional investment chances as growth opportunities, giving the valuation of firms a wholly different point of view [9]. The method also evaluates the predicted values for the company when different strategic options, such as defer, abandon, contract, and expand are given. Through using the real option method, VCs can thus compute a more precise investment value of newly established spinal medical device firms [9]. Nevertheless, the real option methods are strictly quantitative and do not consider qualitative factors [10] or local criteria (e.g., local experts, local universities [11], cultural influences [12], etc.), which are hard to quantify in monetary terms [10]. For example, VCs may consider the firm’s management capabilities, industry–academic cooperation with local universities, product differentiation, external environment, etc. in making decisions. These factors cannot easily be quantified. Multiple criteria decision making (MCDM) methods add important practical contributions in this context, supporting financial decision makers in modeling, analyzing, and evaluating multiple strategies under all decision criteria pertinent to a specific decision instance [13]. The evaluation and selection of investment projects are essential MCDM problems [14].

Thus, the objective of this study is to propose a real option-based hybrid MCDM framework that incorporates the results of real option valuation (ROV) into a hybrid MCDM model, the DANP-mV [15,16], which is composed of two methods: the Decision-Making Trial and Evaluation Laboratory (DEMATEL) based analytic network process (DANP) and the modified VIšekriterijumsko KOmpromisno Rangiranje (VIKOR) method. The proposed analytic framework can be used to manipulate quantitative data, qualitative factors, and local criteria for VCs to evaluate investment projects in spinal medical device firms in general and other type of investment projects in particular. This analytical framework can be divided into six phases: (1) defining qualitative and quantitative constructs and criteria for evaluating projects; (2) defining an analytic framework based on the constructs and criteria defined in Phase 1 by using the DANP; (3) deriving the values of the investment projects using the ROV; (4) grading performance scores of the investment projects based on experts’ opinions; (5) ranking the best investment project(s) by using the modified VIKOR method [17] by incorporating performance scores and results of the ROV; (6) performing sensitivity analysis to determine the influence of modeling parameters on ranking results of alternatives.

This study uses three newly established spinal medical device companies specializing in VCF surgical devices as sample firms and analyzes the performance scores of these three firms using the DANP-mV based MCDM method. Nine senior managers of VC firms in Taiwan were invited to review the criteria for the model. Additionally, these experts confirm the rationality and flexibility of the integrated model at the end of the empirical study process. They also indicate that the integrated model being proposed is useful to VCs for valuation of investment projects not only in spinal medical device firms but also in other firms in different industries. 

The rest of the paper is structured as follows. In Section 2, the related literature on the VCF and the global market, methods of valuating investment projects by VCs, and investment decision as an MCDM problem are reviewed. In Section 3, the research methods, including the modified Delphi method, DANP method, the ROV, modified VIKOR, and sensitivity analysis are introduced. In Section 4, an empirical study based on VCF medical device companies will be introduced to demonstrate the efficacy of the real option MCDM method. In Section 5, the confirmation of our analytical results with past studies and advances in research methods will be discussed. Finally, conclusions, managerial and practical implications, limitations, as well as future research possibilities will be offered in Section 6.

## 2. Literature Review

This section first introduces the VCF and the global market. Then, methods for valuating investment projects are introduced. The methods of valuating investments projects and evaluation criteria used by VCs are reviewed. A review of past work on the applications of MCDM methods in performance assessment is constructive for introducing these methods as a process to evaluate the performance of innovation policy.

### 2.1. The Vertebral Compression Fracture (VCF) and the Global Market

A VCF occurs when the block-like part of an individual bone of the spine becomes compressed due to trauma, osteoporosis, cancer, multiple myeloma, osteogenesis imperfecta, etc. The bone fractures of the upper lumbar and lower thoracic spine are the most common problems [18]. After a number of small compression fractures occur, a wedge-shaped vertebra might be created, which can lead to the stooped posture [19], or the dowager’s hump that inflicts many elderly people. VCF can be divided into acute and chronic [20]. Patients with acute VCF suffer sudden back pain and patients with chronic VCF suffer from lost height, a dowager’s hump, abdominal bulge, etc. [21]. Chronic VCF can also reduce lung function and cause multiple respiratory diseases.

In regular healthy patients, VCF occurs when the bones suffer a strong vertical impact which is common in a car accident, a sport injury, or a hard fall [21]. Metastatic tumors should be considered as the cause in patients younger than 55 with no history of trauma or only minimal trauma [21]. Tumors that result from other cancers and enter the bones are called metastatic tumors of the bone. Most commonly, this type of bone cancer comes from prostate cancer, breast cancer, and lung cancer; such a cancer usually spreads to the spine and causes fractures [22]. Multiple myeloma is a cancer formed from malignant plasma cells [23]. When plasma cells become cancerous and grow out of control, they will stop bone marrow from producing the immune-system cells and make the patients porn to infections and diseases [23]. This type of cancer most commonly occurs in the spinal cord, and 55–70% of these patients will have fractures [24]. 

Currently, vertebroplasty, a minimally invasive surgery through a small puncture in the skin instead of an open incision, which usually takes about 1 hour to complete [25]. There are two treatments in the market, namely percutaneous vertebroplasty (PVP) and percutaneous kyphoplasty (PKP) [26,27]. Before PVP was invented, the traditional way of treating VCF was chronic bed rest [28], medical treatment with pain medication, wearing protective gear, etc. These treatments could ease systems, but they could not help reshape and collapse the spinal cords. PVP is by far the safer and proper medical treatment for VCF. PVP is the earliest treatment. PVP usually involves a percutaneous injection of the cement, polymethylmethacrylate (PMMA), into the vertebral bodies to treat and ease the pain [29]. However, this treatment will not replace the fractured location; instead, it cures the deformation and stabilizes the collapsed locations. As a result, after this treatment, the damaged location is not changed. It is also difficult to estimate the injection volume of the bone meal and the injection location. PKP is an improvement in the medical procedure for existing PVP. The PKP utilizes an inflatable balloon to create space between the bones before the bone is injected to treat and ease the pain [30]. The finished PMMA is harder and stronger than the original bone [31], and thus the reset spine will not compress anymore. The procedure also treats the deformation and collapsed location. 

VCFs often happen to elders and women after menopause. Statistics show that one in three women and one in eight men over 50 years of age have osteoporosis while the most influential risk factor for a VCF is preexisting osteoporosis [32]. According to Alexandru and So [33], about 25% of all postmenopausal women in the US get a compression fracture during their lifetime. Those males who are older than 65 years old are also at an increased risk of compression fractures; however, their risk is significantly fewer than that of women of the same age [33]. The death rate for women with one or multiple fractures is 1.23 times higher than for healthy women [34]. 

According to the statistics by the Allied Market Research [35], currently, North America is still the largest market of VCF devices. Approximately 1.5 million VCFs occur every year in the United States [36]. The annual medical expenditure of treating these injuries is roughly about $746M USD [36]. The Asia-Pacific market is emerging rapidly, with a CAGR of 6.7% [35]. Improving medical infrastructure and increasing medical care spending in emerging economies like India and China are the major drivers for rapid growth. Thus, the major VCF players, which include Medtronic, Depuy Synthes, Stryker, CareFusion Corp., etc. [37], are developing the emerging markets aggressively by adopting product launch strategies.

### 2.2. Methods for Valuating Investment Projects 

VCs usually use DCF methods or the method of “comparables” for valuations of investment projects [38]. Due to some drawbacks of DCF methods, Myers [4] proposed the real option method. Thus, VCs also use real option method to evaluate an investment project. Following, the DCF methods and the real option method will be reviewed in Section 2.2.1 and Section 2.2.2, respectively. 

#### 2.2.1. Discounted Cash Flow (DCF) Method

To evaluate the feasibility of investment projects, VCs estimate expectations of cash flows. VCs then use DCF methods with an appropriate discount rate to determine the value of an investment project. The traditional DCF evaluation methods include NPV, IRR, and others. However, all these traditional methods assume cash flows under an expected scenario and manager commitment to a certain operating strategy throughout the entire time horizon of the projects. These methods will thus not be to take flexible value into consideration. The traditional NPV method is not able to reflect an ever-changing industrial environment. Thus, the NPV method can lead to incorrect evaluations of the investment proposals. The pros and cons of these DCF methods are summarized in the Table 1 below. 

#### 2.2.2. Real Option Method

The Real Option method adopts the ideas of financial options for real assets. The Real Option method is particularly superior when evaluating investment projects with high-risk and high-potential return on investment (ROI). The Real Option method takes the value of flexible strategies into consideration during the managerial decision-making process. The Real Option pricing mechanism was first proposed by Black and Scholes [39] and was mostly used for products with financial options. Myers [8] was the first person to apply the real option method to the evaluation of investment projects. When using the real option method, the drawbacks of the tradition NPV method can be eliminated. The actual value of the investment proposals can more precisely be calculated. This process will not only provide an important piece of information on strategy decision-making to the chief executive officers (CEOs), but also help VCs to obtain an accurate value of the investment proposal.

Myers [40] demonstrated that the traditional evaluation methods always underestimate or ignore factors that might potentially promote the future growth of the company. In other words, they may ignore some factors, which currently might not represent an immediate profit, but might have important impacts on the future of the investment projects. These values cannot be truthfully represented in the NPV method [40]. Copeland and Antikarov [41] demonstrated that the real option method helps managers view future risks as an opportunity for the capital budget and make proper adjustments under different circumstances of the market, such as deferring, expanding, contracting, and abandoning so as to further elevate the value of the specific investment project. 

Trigeorgis [42] categorized real options into the following nine categories (See Table 2): (1) Option to Defer Investment: When the result of the current value of the expected investment profit after deducting the initial development costs is positive, that result represents a positive opportunity value. On the contrary, if the result is negative, the execution of the project should wait until the result turns positive. An investment project similar to this one should have an American Call. (2) Time-to-Build Option: During the staged investment, the amount of capital investment for the next stage depends on its result from the prior stage. In other words, each stage deeply affects the execution or non-execution of the next stage. Because of the nature of one option for each stage, this kind of investment would have multiple American Calls. (3) Option to Expand: When market situations become more favorable than projected ones, managers can expand the scale of production or accelerate the production rate. (4) Option to Contract: When market conditions become weaker than expected, managers will reduce resource utilization or the production scale. (5) Option to Shut Down and Restart Operations: High fluctuation of the market can result in cash flow on the investment profits lower than variable costs. When considering the marginal benefit, managers might pause the investment project and wait until the situation gets better to restart the investment project. In other words, depending on the market situation; it might pause or restart the investment project. (6) Option to Abandon: Recession might force the company to proceed with the project when the profit is higher than the remaining value of the assets. In the other hand, abandonment is taken when the opposite situation happens. On other words, the company will cash out the project to make up for the company’s loss. (7) Option to Switch: When the circumstances change in reality, the investment project will undergo adjustment or a switch of strategies. (8) Growth Options: When the economic boom is on the rise or expanding, in order to increase the growth of the investment project in the future, the manager will need to execute other investment projects to obtain growth of the option value. (9) Multiple Interacting Options: Investment projects usually have multiple options. The total value of these options is not equal to the sum of all the individual options. There will be interaction and affection among multiple options. How these options interact with each other depends on the nature of the options. 

Amram and Kulatilaka [65] categorized the formula for the evaluation methods of real option as follows: (1) Partial differential equations (PDEs): A mathematical equation that connects the value of the real option to the asset value of the target and can be represented by Black and Scholes [39]. (2) Tree method: The tree method will predict the maturity value of the target asset and push back step by step to find the related valuable strategies. The tree method can be represented by the binomial option evaluation method developed by Cox et al. [66]. (3) Simulation method: This method simulates the possible situations and presets the path of any variations accordingly. The value variations of the target asset can thus be predicted. The current value of the real option can be derived by averaging the discounted maturity value. The Monte Carlo Approach was first used in the real option evaluation method in 1977 and was introduced by Boyle [67].

According to Trigeorgis and Mason [51], the real option method can overcome the drawbacks of the NPV method by utilizing the expanded NPV method, which includes both the advantages of the traditional NPV method and the value of the real option method. The value of the expanded NPV method is equal to the sum of NPV and real option value [51]. 

Albeit the NPV method is still regarded as an important evaluation tool for managers, adopting the real option method offers the possibility to estimate the value of the investment project more precisely than NPV. Further, the flexibility of management can be much higher. Therefore, numerous methods and approaches can be applied to evaluate a financial option. These methods include the closed-form equations (e.g., the Black-Scholes Model), the Monte Carlo simulation methods, lattices (e.g., binomial trees), variance reduction techniques, PDEs, etc. [68]. Nevertheless, the binomial lattice tree remains the most popular technique [68]. 

The binomial model, proposed by Sharpe [69] and formalized later by Cox et al. [66], is an evaluation method for option values. The binomial model decreases the probabilities of price changes and eliminates the probability for arbitrage by assuming a completely efficient market. The model evaluates an option at each point in the specific timeframe. The binomial model also takes a risk-neutral channel into consideration and supposes that underlying prices of security can either increase or decrease only until the expiration date of the option. 

The binomial option pricing model is also called a binomial tree. A graphical indication of its potential intrinsic values is an option which might be taken at different nodes or periods. The values of the option rely on the underlying asset. Therefore, the value of the option at any node depends on the possibility of whether the price of the underlying assets at any given node will either decrease or increase. A diagram can be used for strategic decision-making, evaluation, or possibility estimation. The diagram initiates at a single node with divisions spanning out to other nodes, thereby indicating mutually exclusive outcomes.

### 2.3. Investment Decision as a Multiple Criteria Decision Making (MCDM) Problem

According to Tyebjee and Bruno [70], the investment evaluation process for VCs is divided into the originating, screening, evaluating, and structuring of deals, as well as post-investment activities. In the deal origination organization stage, a VC considers the potential investment projects. At the deal screening stage, each VC will screen the investment projects by setting up screening rules to fit its investment strategies and the preferable industries to find the proper fit quickly. In the deal evaluation stage, VCs assess the expected returns associated with the risks based on the business plans provided by the firms that need funds. DCF methods for computing investment valuation include NPV and IRR. Nevertheless, the traditional NPV method, which does not consider operation flexibility and changes in strategy, is far from the reality. Thus, numerous researchers have suggested adopting the real option method to valuate investments [10]. However, real option methods are traditionally designed for financial option valuation, which relies on assumptions, such as random walk, that may fit stock markets but are definitely a poor match with the reality of R&D project evaluation [71] or the valuation of new technology ventures in general, and the new ventures for medical devices in particular. In reality, the cash flows of R&D projects are affected by management actions, and they are seldom random [71]. Further, the real option methods are strictly quantitative. However, the evaluation of an investment project may also contain qualitative factors [10] or local criteria (e.g., local experts, local universities, etc.) [11], which are hard to quantify in monetary terms [10]. For example, VCs may consider the firm’s management capabilities, industry–academic cooperation with local universities, product differentiation, external environment, etc. in making decisions. These factors cannot easily be quantified. Multiple-criteria decision systems add important practical contributions in this context, supporting financial decision makers in modeling, analyzing, and evaluating multiple strategies under all decision criteria pertinent to a specific decision instance [13]. So, the evaluation and selection of investment projects are essentially MCDM problems [11,14,72].

Thus, various scholars have tried to integrate the real option method with MCDM method(s) to solve financial decision problems. For example, Tolga [73] proposed a fuzzy multi-criteria R&D project selection methodology based on the hierarchical fuzzy technique for order preference by similarity to ideal solution (TOPSIS), which includes a fuzzy real options valuation model. Angelou and Economides [10] combined the real option method and the analytic hierarchical process (AHP) into a common decision analysis framework, providing an integrated multi-criteria model for prioritizing information and communication technology (ICT) business alternatives [10]. Tolga, Tuysuz, and Kahraman [74] proposed a fuzzy real option value-integrated fuzzy analytic network process (ANP) method for location selection problems. Restrepo-Garcés, Manotas-Duque, and Lozano [75] proposed a hybrid method composed of techniques of AHP, the TOPSIS, real option analysis, the Monte Carlo simulation, and the binomial method to select the most adequate technologies for electricity self-generation in shopping centers. Recently, [76] integrated real options analysis into an MCDM framework in order to evaluate and holistically rank a portfolio of multiple firms’ projects.

### 2.4. The Evaluation Criteria of Investments Projects for Venture Capitalists (VCs)

In the past three decades, scholars have tried to propose evaluation and analytic frameworks to evaluate and select target firms for investment. According to Fried and Hisrich [77], the earliest investigation of the VCs’ decision process was a study of VCs in Pittsburgh by Wells [78]. The model proposed by Wells [78] was revised and then expanded later by Tyebjee and Bruno [70]. Numerous works have proposed criteria for evaluating possible investment projects by VCs. Typical cases include works by Tyebjee and Bruno [70], MacMillan et al. [79], and MacMillan et al. [80]. These works adopted comparatively easy questionnaires to survey experts from VCs to order the criteria based on to their importance. This methodology was criticized later for some aspects by Sandberg et al. [81]. Hisrich and Jankowicz [82] and Hall and Hofer [83] studied the topic by adopting totally different methods. However, very little work has investigated the whole evaluation procedure regarding the decision made on an investment project. Riquelme and Rickards [84], however, proposed a self-explained, hybrid conjoint analysis model to help evaluate and select new ventures for investment in a VC scheme. By summarizing these past works, the evaluation criteria for investments projects for VC can be summarized in Table 3 as the basis for developing the ROV-based MCDM analytical framework in this current study. 

### 2.5. Research Gaps

Based on the above literature review results, five research gaps can be identified. First, the real option methods are strictly quantitative. However, the evaluation of investments may also contain qualitative factors, which cannot be quantified in monetary terms [10]. Second, though some scholars ([10,74,75,76]) have tried to integrate the real option method and the MCDM framework during the past few years, the traditional MCDM methods like AHP, TOPSIS, etc. assumed independence between criteria. Meanwhile, the adoption of the ANP with ROV requires assumptions about the structure of the decision problem, which are far from realistic. An analytical framework that could overcome traditional assumptions would be more realistic. Third, though modern DEMATEL- and DANP-based MCDM methods have been widely adopted in various applications, based on the authors’ very limited knowledge, such methods have seldom been integrated with the real option method. However, such integration is very meaningful, especially in terms of crossing the research gap in dealing with the traditional problem of ROV-based analyses in handling qualitative factors as well as the AHP-based integrated framework. Fourth, VCs actually evaluate investment problems by considering numerous qualitative criteria, which are hard to evaluate by real option methods. Thus, an integrated framework considering both the quantitative valuation of an investment project and the qualitative decision criteria will be very reasonable for the investment decisions. Finally, the adoption of the MCDM framework on the evaluation of investment projects of medical devices in general, and the spinal medical device firms are rare. However, such kinds of investment projects will become daily dominant, especially in the sixth Kondratieff wave [85], where bio/medical technology will be the major driving force for global economic growth. Thus, a novel analytical framework integrating the modern MCDM methods as well as the ROV method will be very important for filling the research gap mentioned above.

## 3. Research Methods

To evaluate and select investment projects in spinal medical firms by adopting real option, valuation-based MCDM methods, this research assesses and confirms the criteria collected by the literature review based on the modified Delphi method. Then, influence relationships among the aspects and criteria are structured using the DEMATEL method. The weights associated with each aspect and criterion will then be derived using DANP. Then, spinal medical device firms will be evaluated using the MCDM-based ROV method. Finally, these firms will be ranked using the modified VIKOR method.

Various MCDM methods can be considered while selecting appropriate methods for solving a decision-making problem. However, the assumptions, weaknesses, or limitations associated with some methods limit their application in the evaluation and selection of an investment project in spinal medical device firms. The statistical methods (e.g., [86,87]) are not so appropriate for evaluation and selection problems. Traditional methods for deriving weights associated with each aspect and criterion, which have already been adopted in the integrated decision framework with real option analysis, e.g., the AHP, always assume independence between criteria, which is against the nature of most decision problems in general, and the specific material selection problem especially. As the aspects and criteria belonging to real-world decision-making problems always influence one another, an analytical framework consisting of DEMATEL and DANP is very suitable [88]. DEMATEL can be used to structure the influence relationships between the criteria. Then, based on the structural model as well as the total-influence matrix derived using DEMATEL, DANP can be introduced to derive the weights associated with the criteria. Finally, the modified VIKOR method, based on the concept of a compromise solution proposed by Yu [89] and Zeleny [90], can overcome the limitations of TOPSIS [91] and determine the best alternative with confidence. Therefore, the proposed DANP-mV method can be used to integrate with the real option method and rank the alternatives.

**Figure 1:**
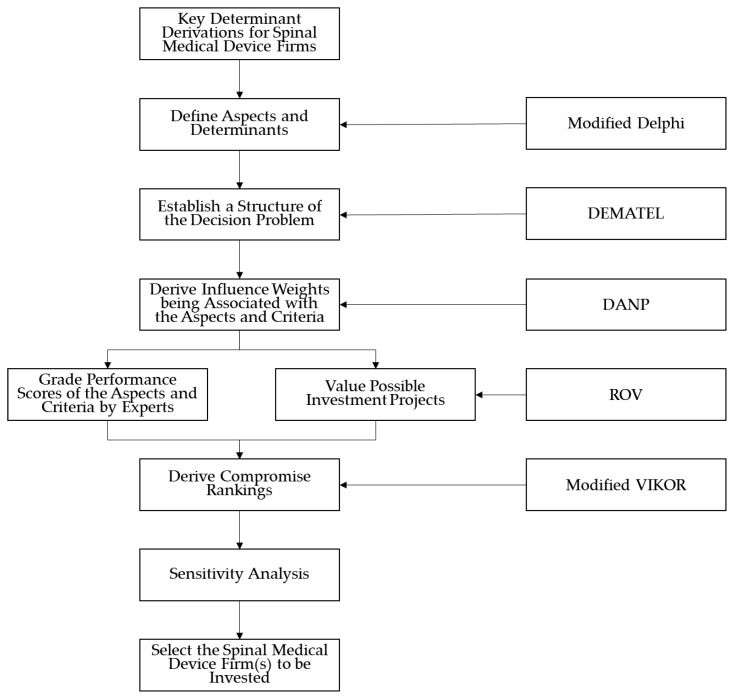
The flowchart of decision-making framework. DEMATEL—Decision-Making Trial and Evaluation Laboratory; DANP—DEMATEL based analytic network process; ROV—Real Option Valuation; VIKOR—VIšekriterijumsko KOmpromisno Rangiranje.

In summary, this decision-making framework consists of five major phases: (1) deriving determinants using the modified Delphi method introduced in Section 3.1; (2) constructing an influence relation map (IRM) by using DEMATEL, introduced in Section 3.2; (3) deriving the influence weights associated with DANP, introduced in Section 3.2; (4) deriving the real option values of the spinal medical device firms by using the ROV methods introduced in Section 3.3; (5) deriving the compromise ranking of the three target firms by using the modified VIKOR method introduced in Section 3.4; and (6) performing sensitivity analysis to determine the influence of modeling parameters on ranking results of alternatives. The flowchart is shown in the following Figure 1. 

### 3.1. Modified Delphi Method

The Delphi method was designed by Dalkey and Helmer [92] to summarize the opinions being provided by respondents on specific topic(s) under investigation. Due to the tedious process involved in manipulating the Delphi, Murry, and Hammons [93] later revised the conventional Delphi method by substituting the open questionnaires then adopted in Delphi by using questionnaires that were carefully reviewed and selected. The questionnaires may come from the literature, experts’ opinions, etc. The major benefits of the modified Delphi method include an easy-reached consensus [94], a high response rate, solid ground of the questionnaires, the reduction of possible biases or errors, assurance of the privacy of the respondents, and providing the controlled results to those who participated in the surveys. 

### 3.2. DANP Method

The DEMATEL method was proposed by Gabus and Fontela [100] from the Geneva Research Centre of the Battelle Memorial Institute in order to convert a complicated world problem into a clearly demonstrated and influential relationship for the root causes of these problems. The major concept of this technique is to define a set of influence relationships based on a network structure. The DEMATEL aims to use matrices to construct direct and indirect influence relationships and assist in identifying the influence strength amongst the aspects/criteria being considered. Unlike the traditional statistical methods (e.g., the correlation analysis or structural equation modelling) which require an especially large size of samples in order to derive the causal relationships among variables based on the correlation variables, the DEMATEL-based method needs only the opinions provided by a limited number of respondents [101] to derive acceptable influence relationships [102]. Please refer to Appendix A of the first author’s earlier work [103] for these detailed procedures.

The major purpose of the DANP is to transform the total influence matrix being derived by the DEMATEL to an unweighted super matrix, and then derive the influence weight associated with each aspect and criteria accordingly. The major goal of introducing the DEMATEL technique is to utilize the graph theory of discrete mathematics to investigate complicated practical problems. The qualitative and mutually influenced aspects of decision-making problems can then be resolved. The DANP can reflect the essence of a decision problem by preventing the loss of any information from the trimming of any influence relationship caused by defining a threshold value and the simplifying of the investigation process by reducing the survey time required for the conventional ANP processes. Please refer to Appendix C of the author’s earlier work [103] for these detailed procedures.

### 3.3. Real Option Valuation (ROV)

The binomial method is an outstanding technique for making financial decisions. This method offers an effective structure wherein alternatives can be evaluated. The method further helps establish a correct and balanced picture of the risks and rewards from a specific choice. To introduce the binomial method for ROV, the analytic procedure is defined by modifying the work done by Kodukula and Papudesu [99]. The steps of the binomial method for ROV are described as follows. 

#### 3.3.1. The Procedures of the Binomial Method for ROV

At first, the input parameters should be identified. The elementary input parameters for the binomial method used to evaluate any type of option include revenue (*sa*), project cost (x), and option life (t). r is risk-free rate corresponding to the option life. The risk-neutral probability is denoted by *p*. σ is the percentage of volatility indicated by the standard deviation of the natural logarithm of the underlying free cash flow returns. δt is the incremental time step to be considered over the option life. The up and down factors, i.e., u and d, can be defined as:

(1) $$u=\exp(\sigma \sqrt{\delta t}),$$

(2) $$d=\exp(-\delta \sqrt{\delta t})=1/u,$$

Here, the units of σ and δt should be consistent. The risk-neutral probability, *p*, is defined as follows:

(3) p=[exp(rδt)−d]/(u−d)

Then, the binomial tree can be built. The asset values at each node of the tree can be derived using the number of selected time increments based on the following procedure.

**Figure 2:**
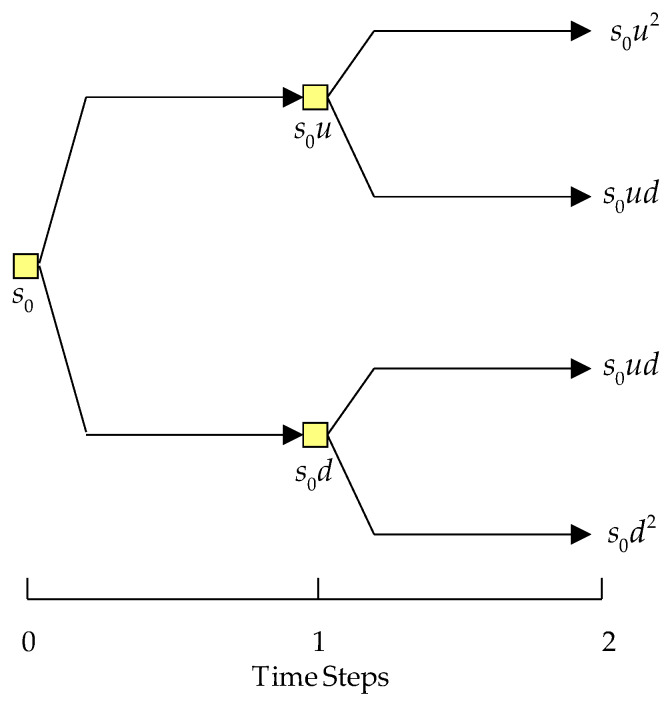
Binomial Lattice. Source: Adapted from Kodukula and Papudesu [99].

The construction of the binomial tree (See Figure 2) for *t* years initiates from the left end of the tree (time zero) and moves to the right by taking one year as the interval. The asset values will then be calculated over the whole lifetime of the option. Assume the value of the asset is $s_{0}$ in the beginning of the investment project that is a spinal medical firm, the asset value will be multiplied by the up factor *u* as $s_{0}u$ and the down factor *d* as $s_{0}d$, respectively, for the first year. Moving to the right end of the binomial tree, the value associated with every node can be derived by using the same approach until the last step. In Figure 2, the upper value at each node represents the asset value at that particular node. 

Finally, the option values associated with every node can be calculated by backward induction from the very end of the right-hand side of the binomial tree; the decision rule is applied at each node. Heading to the left-hand side of the tree, the option values associated with each node can be calculated by introducing the option values from the succeeding nodes, then discounting them using a risk-free rate and the risk-neutral probability factor. This process is repeated until the far left end of the tree is reached. The option value can thus be derived. 

#### 3.3.2. Procedures for Backward Induction

First, the values of the terminal nodes representing the last time step should be derived. Then, the values associated with the intermediate nodes which is one step away from the last time step is derived. The expected asset value for any intermediate node can simply be derived by the discounted (at the risk-free rate) weighted average of potential future option values using the risk-neutral probability. That is, the value at year x of the node $\left(i_{x},j_{x}\right)$ is $s_{0}u^{i_{x}}d^{j_{x}}$, where

(4) $$s_{0}u^{i_{x}}d^{j_{x}}=[p(s_{0}u^{i_{x}+1}d^{j_{x}-1})+(1-p)(s_{0}u^{i_{x}-1}d^{j_{x}+1})]\exp(-r\delta t),$$

while $i_{x}-1\geq 0\;\mathrm{and}\;j_{x}-1\geq 0,$

(5) $$s_{0}u^{i_{x}}d^{j_{x}}=[p(s_{0}u^{i_{x}+1})+(1-p)(s_{0}u^{i_{x}-1}d^{j_{x}+1})]\exp(-r\delta t){,}_{\;}$$

while $j_{x}-1<0,$ and

(6) $$s_{0}u^{i_{x}}d^{j_{x}}=[p(s_{0}u^{i_{x}+1}d^{j_{x}-1})+(1-p)(s_{0}d^{j_{x}+1})]\exp(-r\delta t),$$

while $i_{x}-1<0,$ i+j=x.

The $s_{0}u^{i_{x}}d^{j_{x}}$ value is equal to the discounted value at year *x* by using the risk-free rate as the discount rate. The decision-maker can evaluate what the value-maximizing decisions would be at the nodes that represent the earlier years.

### 3.4. Modified VIKOR

The VIKOR method was proposed by Professors Opricovic and Tzeng [104] to derive alternative(s) with conflicting criteria based on the concept of a compromise solution as proposed by Yu [89] and Zeleny and Cochrane [90]. Indifferent from the conventional TOPSIS method [105,106], which ranks the solutions by averaging the shortest distance to the optimal solution and the longest distance from the worst solution, the VIKOR considers the comparative importance of these two distances and derives a rational compromise solution by adopting the maximum group utility of the majority (as represented by min *S*) and the minimum of the maximum individual regret of the opponent (as represented by min *Q*). The modified VIKOR method further extends the concept of the VIKOR by replacing the shortest distance to the optimal solution with the shortest distance to the aspired solution. The proposed approach can prevent the problem of selecting the best solution from a group of unsatisfied alternatives. For the detailed process of the modified VIKOR, please refer to [17] and Appendix D of the author’s earlier work [103]. Based on the methods introduced above, this work adopted the DEMATEL to construct the decision-making problem and the DANP for the influence weights associated with the aspects and the criteria, and then adopted the modified VIKOR to derive a compromise ranking of the spinal medical device firms. The selected firm(s) are the most suitable ones for investments by VCs.

### 3.5. Sensitivity Analysis

The goal of sensitivity analysis is to determine the sensitivity (or influence) of modeling parameters (criteria weights in this study) on the final results of the decision-making process. In order to assess the impact of criteria weights on the rankings of the alternatives obtained from the modified VIKOR method, five set of experiments were conducted based on the analytic process being proposed by [107]. In the first set of experiment, the weight of each criteria and the ROV aspect ($D_{8}$) is set to the maximum value (i.e., 100%) one by one. The remaining criteria weights are set to 0. This leads to 41 experiments. Then, in the second set of experiment, all criteria weights are set equal, the total criteria weight being equal to 1. In the third set of experiment, each criterion is assigned a random weight. 100 tests were executed. In the fourth set of experiment, only the criteria belonging to one specific aspect are used and given equal weights. All aspects (except for the ROV aspect) are analyzed. In the fifth set of experiment, the utility value v is varied from 0 to 1. This leads to 11 experiments. In the proposed DANP-mV model, the parameter *ν* has been introduced as weight of the strategy of the maximum group utility. By referring to refer to [17,103], the index values of VIKOR can be formulated as $R_{l}=$$v(S_{l}-S^{*})/(S^{-}-S^{*})+(1-v)(Q_{l}-Q^{*})/(Q^{-}-Q^{*}).$ The parameter *ν* plays an important role in the ranking of three investment projects. Generally, the value of *ν* is taken as 0.5. However, *ν* can take any value from 0 to 1. Therefore, it is necessary to conduct a sensitivity analysis on the parameter *ν* for validating the obtained results. Overall, a total of 160 experiments were conducted.

## 4. Empirical Study Results

In this Section, the empirical study case is introduced in Section 4.1. Then, the constructs and criteria being proposed in Section 2.4 and summarized in Table 3 are confirmed by experts in Section 4.2. The ROV for the investment projects is analyzed in Section 4.2. The weights associated with each criterion are derived in Section 4.2.4 using the DANP-mV. Finally, performance scores or the target firms are derived.

### 4.1. Empirical Study Cases

Three emerging Taiwanese companies, S, W, and B, will be the focal firms for the empirical study cases. The VC managements would like to evaluate these three companies as their basis for investment. The background of these three companies is offered below. 

Company S was established in August of 2013 with the current capital of $4.73M USD. Its main product is orthopedic medical equipment, which is mainly used in VCF and includes Vessel-X. Osteo-G, PKP, PVP, etc. In 2015, the company accounted for 40% of the total market sales, and other regions had 60%. The new product for the company, Vessel-X, was developed using the existing technology of PVP and PKP. Thus, doctors can easily learn and operate that new technology. The medical risk is low, and the efficacy of the medical services is improved. Vessel-X has been granted a sales license by the Governments of Taiwan and China. The firm is now in the process of applying for 510(k) from the Food and Drug Administration (FDA) in the United States.

Company W was established in December of 2009. The firm is a spin-off of the Industrial Technology Research Institute (ITRI), in Taiwan and the INTAI Technology Co. The W company is dedicated to the research and production of a wide variety of high-end, implantable, Class II, and Class III medical devices that include bone graft substitutes, spinal fixation systems, an interbody fusion system, and minimally invasive surgical instruments. The R&D strategy of this company emphasizes designing products with safety and great efficiency to meet the needs of clinicians. Their Good Manufacturing Practice (GMP) plant can manufacture and supply the best and latest medical devices and market their own product lines. The product line for the bone graft substitutes has accounted for 20% of their market share in Taiwan. Nowadays, many of their products have received certifications and licenses from the Governments of Europe, United States, China, South-East Asia, and more. Many of their products have also been certified by the EU. 

B company was established in 2009 and started to sell their own product, the ReBorn Essence in September 2009. Based on the experiences of the foundry and the manufacturer of medical implants for more than 20 years, the company has become the manufacturer of competitive orthopedics medical equipment. Their product lines include but are not limited to spinal cord implants, orthopedic materials, and surgical instruments. Based on the 7 patents granted by the world’s major economies, the company’s goal is to provide products and facilitate equipment to simplify the surgery procedure and optimize the quality of medical treatments. Since company B’s product was approved in September of 2014, the firm has attended numerous global exhibitions of medical equipment and received awards, e.g., the 10th Industrial Innovation Award granted by the Taiwanese Government, and many other positive reactions. The firm expects to develop their own brand-name products to increase its domestic market share and also market their products globally. 

Due to their great potential of growth, the three spinal surgical device firms above have become the investment targets of VCs that focus on high growth industries. Before that investment(s), VCs should evaluate and select the appropriate firm(s). The analytic procedure that uses the proposed ROV based MCDM framework will be demonstrated in the following sub-sections.

### 4.2. The Procedure of Empirical Study

The DANP-mV based method will be able to evaluate the actual value of an investment proposal precisely and evaluate the qualitative factors concurrently. To evaluate the investment projects, the research will first confirm the constructs and criteria in Section 4.2.1 using the modified Delphi introduced in Section 3.1. Then, the influence relationships between the aspects and criteria will be derived in Section 4.2.2 using the DEMATEL introduced in Section 3.2. Performance scores of the three focal firms will be graded in Section 4.2.2 based on the constructs and criteria being summarized in Table 3 by experts from the VC industry. After that, the value of the investment (*D*_8_) being associated with the firms will be valued by using the proposed ROV method being introduced in Section 3.3. Then, the valuation results derived by using the ROV will be fed into the DANP-mV based MCDM framework as the value of an aspect. Finally, the weights g associated with each aspect and criteria will be derived using the DANP introduced in Appendix C of the author’s earlier work [103]. The VIKOR values and the compromise ranking for the three focal firms will be derived based on the ROV and the MCDM integrated framework. 

#### 4.2.1. The Criteria Confirmation Using the Modified Delphi Method

To derive the aspects and factors for evaluating and selecting the investment projects of spinal medical device firms, the modified Delphi method is applied to confirm the criteria based on the experts’ opinions. Nine senior managers from the Taiwanese VC firms (see Table 4) were invited to provide their opinions. The opinions provided by these experts are summarized in Appendix A. Based on the results of a modified Delphi analysis, all of the requirements are recognized as suitable or evaluating the investment projects.

#### 4.2.2. Decision Problems Structuring and Performance Score Grading

**Figure 3:**
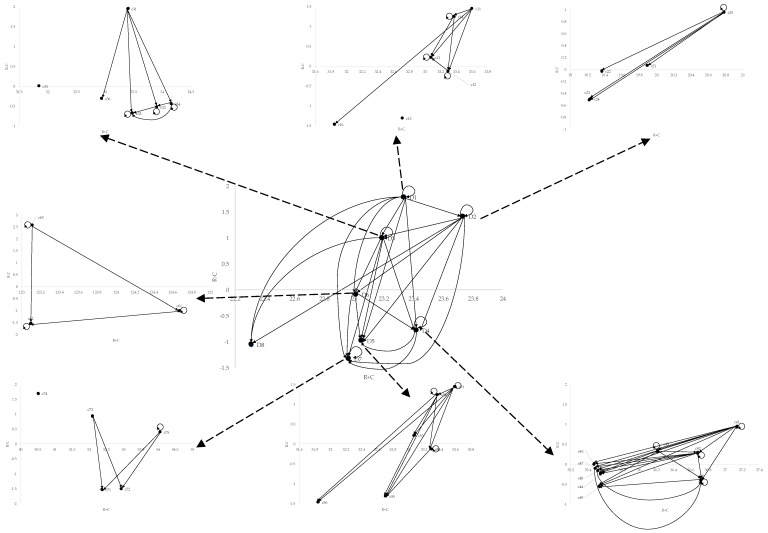
The Causal Relationship Structure of Each Dimension and Its Criteria.

This current research collected expert questionnaires from nine VC experts. The experts come mainly from Taiwanese VC firms are at least Deputy Managers. Based on these experts’ opinions, the causal relationship and their weights versus the criteria are derived as follows. The aspects and criteria being derived are based on literature review results (see Table 3) that were confirmed by experts. Then, the initial direct influence matrix *D* was obtained based on the opinions of nine experts. The experts were asked to indicate the direct effect that they believe a factor *i* will have on factor *j*, as indicated by $d_{ij}$. The matrix reveals the original interrelationships with these criteria based on the viewpoint of each individual expert. Then, the direct-influence matrix *D* can be derived based on Equation (A2) of [103] as a normalized matrix *N* (refer Table A2 of Appendix C). The total-influence matrix *T* (refer Table A3 of Appendix C) and the causal relationship (Figure 3) are derived by using Equation (A3) of [103]. In matrix *T*, the (*i*, *j*) element $t_{ij}$ denotes the direct and indirect influences of factor *i* on factor *j*. 

Further, the causal relationship can be structured based on the total relationship of matrix *T*. The influence relationship will be defined based on the threshold value. The values of ${(r}_{i}{+c}_{i})$ and ${(r}_{i}{-c}_{i})$ are demonstrated in Table 5, while the influence relation map (IRM) is demonstrated in Figure 3. The ${(r}_{i}{+c}_{i})\;$ value indicates the strength of the influence associated with the $i_{th}$ criterion by summarizing the influences of that criterion on other criteria as well as the influences of all the other criteria on the $i_{th}$ criterion. The ${(r}_{i}{-c}_{i})$ value is the degree of the central role that factor *i* plays in the decision problem. If ${(r}_{i}{-c}_{i})$ is positive, then the criterion mainly dispatches influence on the other criterion; and if ${(r}_{i}{-c}_{i})$ is negative, then the criterion mainly receives influences from other criteria. 

Thus, the influences among the criteria and the ranking of the criteria can be derived accordingly. At first, the potential growth of the market impacts the following criteria: The investment project’s scale and investment strategies, the estimated ROI ratio of the investment projects, the investment projects that have a shorter recall period (about 2–3 years), the potential of being merged and the acquired, the withdrawal mechanisms of the investment project. Second, market demand impacts the following criteria: The investment project’s scale and investment strategies, the estimated ROI ratio of the investment projects, the investment projects that have a shorter recall period (about 2–3 years), and the withdrawal mechanisms of the investment project. Third, the market acceptance influences the following criteria: Patent protection, unique products and technologies, a competitor’s entry barriers, great growth potential or operation profit, technical level of the products, gross profit of the products. Market acceptance impacts the investment project’s scale and the investment strategies, the estimated ROI ratio of the investment projects, the investment projects that have a shorter recall period (about 2–3 years), the potential of being merged and acquired, and the withdrawal mechanisms of the investment project. 

Based on these analytical results, the top ranking criteria are as follows: Potential growth of the market, market demands, market acceptance, the estimated ROI ratio of the investment projects, the investment project’s scale and investment strategies, the withdrawal mechanisms of the investment project, the investment projects that have a shorter recall period (about 2–3 years, the potential of being mergers and the acquisition of the investment project, the gross profit of the products, and competitors’ entry barriers.

After deriving the causal relationship, the criteria weight can be derived by using the DANP. First, the total-relation matrix *T* was taken as the input to derive the influence weights versus the criteria. The criteria weights were normalized using the influence matrix $T_{D}$. Based on the normalized total-influence matrix $T_{D}$, the unweighted supermatrix *W* can be derived by using Equation (A14) of [103]. These total influence values will then be used to derive the weighted supermatrix. Finally, by raising the power of the non-weighted supermatrix to infinity using Equation (A15) of [103], the influence weights versus each aspect and criterion (see Table 5) can then be derived.

#### 4.2.3. Derivations of ROVs Using the Binomial Method

This research uses the binomial method to derive the value of the ROV. The process was introduced in Section 3.3. First, the financial value for input for the binomial method is derived from the experts’ opinions for the investment projects of the three companies. They are represented by the numbers in the initial investment and the possible cost of the project costs (*x*). Based on the experts’ opinions, the initial investment for S company is $100,000 million USD dollar; that for W company is $90,000 million USD dollar, and that for B company is $80,000 million USD.

Then, the values of the real options by using the ROV for the S, W, and B companies can be derived by using the following process. The option life is 5 years. The assumed volatility of the asset value (*σ*) by assumption is 30%. The risk-free rate corresponding to the option life (*r*) is assumed as 5%. Finally, the incremental time step to be considered over the option life (δt) is 1 year. 

After the parameter definition, the option parameters *u* and *d* can be derived using Equations (1) and (2), respectively, as 1.350 and 0.741. Then, the risk-neutral probability, *p*, can be derived as 0.510 using Equation (3). Then, the binomial tree can be constructed based on the derivations of the asset values of each node. So, by starting with $s_{0}$, the asset values associated with the next time step can be derived by multiplying the up (1.350) and down (0.741) factors, respectively. 

**Figure A1:**
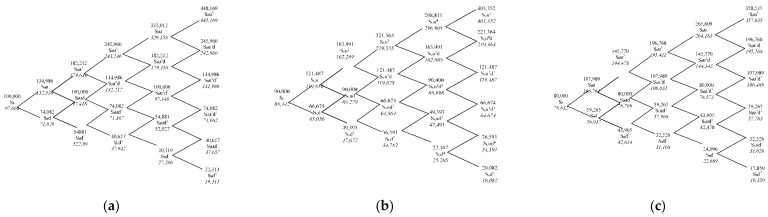
Option Value Binomial Tree for company (**a**) S, (**b**) W, and (**c**) B. Remarks: All numbers are thousands USD. Top numbers are asset values. Bottom italicized numbers are option values. Option to invest is exercised at notes where the option value is not zero.

The option value on each intermediate node is derived using the procedures of the backward induction introduced in Section 3.3 and the Equations (4)–(6). These procedures are illustrated in Figure A1a–c in Appendix B for S, W, and B, respectively. All option value on each node is the discounted value by using the risk-free rate of 5% as the discounted rate. Each node represents the maximum value of investment at the specific point in time or waiting until the following time period. At each node, the investors can choose whether to invest or wait until the next time period before the option terminates. 

For a 5-year option life, the ROV at the fifth year can be derived using Equations (4)–(6) as follows:

$$\mathrm{ROV}=\left[p\left(S_{0}u^{5}\right)+\left(1-p\right)\left(S_{0}u^{4}d\right)\right]\times \exp(-r\delta t)$$

The ROV is equal to the discounted value derived using the risk-free interest rate of 5% as the discounted rate. Finally, the values of the real options being derived by using the ROV will be normalized. The normalized values will then be multiplied by 5 to fit the 5-Point Likert Scale (refer to Section 3.3.) being leveraged by using the MCDM framework in Section 4.2.1. The ROV for the three firms are summarized in Table 6. 

#### 4.2.4. Derivation of the Compromise Ranking

After the derivations of weights versus each aspect and its criterion, the three firms are ranked using the performance scores as graded by the experts and the influence weights as derived using the DANP method. Based on the average performance scores graded by the nine experts (Table 7) by integrating the ROV results (Table 6) derived in the prior Section 4.2.3 as the performance scores of $D_{8}$, the compromise ranking results are derived using the modified VIKOR method in Section 3.4. In the modified VIKOR method, the positive ideal solutions are replaced by the aspired level, which is 5.000 of the five-point Likert scale adopted in this work. According to these analytic results, the performance scores of the three firms S, W, and B are 0.000, 0.494, and 1.000, respectively. Therefore, firm S is the most appropriate target for investment since the synthesized gap of the constructs and criteria to the aspired level is the least one.

#### 4.2.5. Results of Sensitivity Analysis

**Figure 4:**
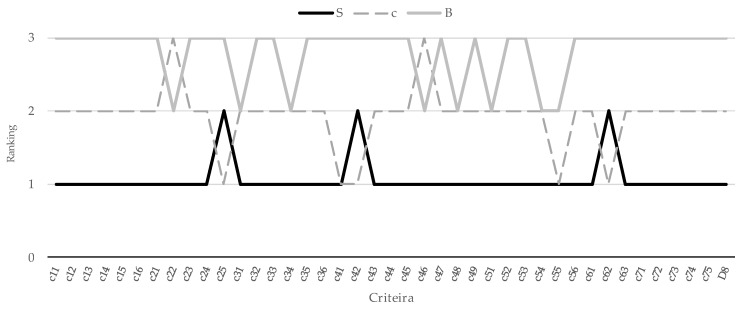
Results of the Experiment 1 of the Sensitivity Analysis.

Based on the procedure of sensitivity analysis being proposed in Section 3.5, five set of experiments are analyzed. The results of sensitivity analyses for the 160 experiments are demonstrated in Appendix D. The firm S is rated as the most appropriate target for investment for 157 times. The rankings were changed only when the weights of criteria *c*_25_, *c*_42_, and *c*_62_ were set to the maximum value, 100%, respectively, while the remaining criteria weights being set equal to 0. Meanwhile, the values for firms S and W are equal in the first set of experiment. (Refer Figure 4 for results of the first set experiments, where the ranking changes.) This result shows that the obtained results of the proposed approach are robust and reliable. 

#### 4.2.6. Confirmation of Analytical Framework and Results by VC Experts

At the end of the empirical study process, the nine VC experts (refer Table 4) were invited again to confirm the rationality of the ROV-based framework, the analytical results, and the feasibility of the proposed framework. Currently, most Taiwanese VCs are still using traditional valuation methods like IRR and NPV. Even the ROV-based methods have rarely been adopted. Considering the drawbacks of the traditional IRR and NPV methods as well as the limitations of the ROV-based methods in manipulating qualitative factors or local criteria, which are hard to quantify in monetary terms, these experts agreed that the aspects and criteria are reasonable. Further, the senior managers from VCs also agreed that the MCDM framework can be feasible with the assistance of MCDM experts in the manipulation of the research methods or expert systems like Super Decisions [108]. Finally, all nine experts indicate that the integrated model proposed in this study is useful for VCs to evaluate investment projects not only in spinal medical devices firms but also in other firms in different industries.

## 5. Discussion

In this research, three spinal medical device firms were evaluated using the real option and DANP-mV based MCDM method and the VC’s viewpoints. While a suitable firm was selected, that rationale will be discussed in the following subsection. In Section 5.1, the managerial implications are discussed. The consistencies between our analytical results demonstrated in Section 4 and the prior studies were confirmed. In Section 5.2, mutual influences of the most important criteria are discussed. Advances in research methods are discussed in Section 5.3. Finally, both research limitations and directions for future research to take are presented to end this section.

### 5.1. Managerial Implications 

According to the empirical study results, weights associated with the aspects, dimensions 7, 4, 5, and 8 are the more important ones. The Firm S outperformed the other two firms in these four aspects. Therefore, the firm was selected as the most suitable target for VC investment. These aspects represent the business model ($D_{4}$), nature of the investment ($D_{7}$), the product ($D_{5}$) and value of the investment ($D_{8}$). For example, Mason and Stark [109] argued that the business model must make sense. Further, according to the work by Chen et al. [110], the major reasons for VCs’ funding decisions are: (1) a reasonable business model ($D_{4}$); (2) creative product(s) ($D_{5}$); (3) an enthusiastic management team with strong backgrounds, (4) a fantastic market, and (5) a feasible business idea ($D_{4}$) which can potentially generate profits. Witt et al. [111] argued that whether a set of standard performance measurement criteria can be well applied to new ventures depends on the industry and the business model ($D_{4}$). This argument is also consistent with our analytical results. 

Although the firm was selected, most of the criteria did not achieve the aspired level. Therefore, after the investment, the VC management can consider strategies to enhance the operation of the firm. Based on the analytic results derived by DEMATEL, the aspects $D_{2}$ (professional competencies of the management team) and $D_{1}$ (nature of the management team) were the most dominant ones, with the highest ${(r}_{i}{+c}_{i})$ and ${(r}_{i}{-c}_{i})$ values. Thus, the target firm can further be enhanced by strengthening the professional competencies of the management team and improving the nature of the management team. Then, the competencies and nature of the management team can achieve the aspired level. 

These analytical results are consistent with past efforts. The pivotal study by MacMillan et al. [79] regarding to the criteria being used by VCs for evaluating novel proposals for new ventures argued that half of the most dominant criteria are about the personal characteristics or experience of the entrepreneurs. According to Muzyka et al. [112], the critical factors being adopted by VCs when evaluating possible investment projects include: leadership of the lead entrepreneur and the management team; acknowledged practical expertise of the team; and achievements of the lead entrepreneur and the management team. All were concerned with the characteristics of both the entrepreneur and the team [113]. Meseri and Maital [114] argued that the quality and motivation of the leadership team are critical elements for the final success of any new venture. The competencies of the management team can be regarded as the key decision factors for a new venture’s decision-making and final success [115,116]. According to Hsu [117], founders with previous successful experiences with new ventures offer probably clear signs of their entrepreneurial quality and expertise. Kollmann and Kuckertz [118] argued that the impacts of a management team’s quality on the performance of start-ups have been verified by numerous empirical results. 

According to Muzyka et al. [112], the top five criteria selected by VCs in their investment decision-making encompass (1) sustained competitive advantage; (2) sales and marketing capabilities of the team; (3) administration capabilities of the team; (4) the capability to realize capital gains; and (5) the degree of understanding regarding the product and its market(s). That is, a good management team can improve a medium type of product. At the same time, a poor management team may not be able to cope with an excellent product. This argument is consistent with our research results. The product is an important criterion. However, the product criterion is influenced by the capability of the management team. Following, the most important aspects and criteria will be discussed further. The consistency between the analytic results and past works are also provided. 

#### 5.1.1. The Aspect of Nature of the Investment Project $\left(D_{7}\right)$ and Criteria

The aspect of nature of the investment project $(D_{7}$) includes five almost equally important criteria. Shorter payback period (about 2–3 years) (*c*_73_) and withdrawal mechanisms for the investment project (*c*_75_) are the more influential ones. These two criteria influence investment scale and strategies (*c*_71_) and the estimated ROI ratio of the investment projects (*c*_72_). The potential of being merged and acquired (*c*_74_) is independent of the others. 

At first, the main investment goal of most VCs is high expected return and shorter payback period [119]. This is consistent with our analytic results in that a shorter payback period (*c*_73_) is an important criterion. Further, according to Carter and Van Auken [120], VCs without any business background are characterized by (1) investing in earlier stages of a firm; (2) requiring a shorter payback time (*c*_73_); and (3) making more investments than those VCs with business background do. The argument by Carter and Van Auken [120] is consistent with the influence relationship being derived, namely the shorter payback period (*c*_73_) influences the investment scale and the strategies (*c*_71_). 

According to the survey on business angels by Hindle and Lee [121], about 27% of the respondents regarded the exit mechanism (*c*_75_) as an important factor and about 53% of the respondents regarded the payback period as important [121]. For low profit firms, the efficiency of the exit mechanism (*c*_75_) is relatively more important than the ability of the VC to extract rents; hence there exists a cut-off level for expected profits such that firms with expected profits above this level will be exited using IPOs and firms below that will be exited using trade sales [122].

First, the research result for the withdrawal mechanism (*c*_75_) is consistent with earlier works [123]. Uncertainty plays a dominant role in exit and withdrawal decisions based on the classical real options theory [42,124,125,126]. According to Gravel [127], VCs often require a mechanism whereby they may withdraw at a set date for a set price to ensure a predetermined minimum return. According to Cumming [123], the option to withdraw (*c*_75_) or deliver a put option can be an important strategy for VCs when they make decisions about a sequential investment that is under uncertainty [42,128]. According to Cumming [123], keeping the withdraw option alive enables a firm to capitalize, i.e., leave money for the upside possibility of a project. Exercising this option lets the firm limit downside risks [42,123,128]. 

According to Li and Chi [126], intervention in the withdraw from investment project(s) changes the profit and loss distribution into a favorable asymmetrical form [50,61]. The arguments by Li and Chi [126] are consistent with our analytic results in that the withdrawal mechanisms for the investment project (*c*_75_) influence the estimated ROI ratio for the investment projects (*c*_72_).

#### 5.1.2. The Aspect of Business Model ($D_{4}$) and Criteria

The aspect of business model ($D_{4}$) has been recognized as important in this work. The analytic results are consistent with the empirical results of prior studies. According to Zacharakis et al. [129], VCs should be able to interpret information at the environmental, business model ($D_{4}$), and team level. Chen et al. [110] argued that one of the main reasons for an investment decision is a reasonable business model ($D_{4}$). Apparently, the business model ($D_{4}$) is a very important aspect that influences the investment decisions made by VCs.

According to Wiltbank et al. [130], the evaluation criteria for investment decisions of new ventures can change according to the stages in the lifecycle of a venture. Investors are very careful about the stage of those opportunities in which they invest [131]. At the earlier stages of the lifecycle, the challenges are more significant when evaluating the business model ($D_{4}$), the real market opportunity, and the growth possibility [132]. According to Kaplan and Strömberg [133], in at least one-third of these investments, VCs were attracted by the product, the technology, the strategy, the business model ($D_{4}$), the high likelihood of customer adoption, and the favorable competitive position.

The research results by Frank et al. [134] also offered several interesting insights. For example, the evaluation *t* of business models ($D_{4}$) (e.g., the work by Amit et al. [135]) could depend on experience effects. Whereas novel VCs may look at independent components of business models ($D_{4}$) (e.g., transaction efficiency), practiced VCs may emphasize the fit of the various components more and thus may have better understanding of the total value creation possibility of the proposed venture.

Among the criteria belonging to the aspect of business model ($D_{4}$), value proposition (*c*_41_) is the most influential. The value proposition (*c*_41_) affects customer relations (*c*_44_) and the main source of profit (*c*_46_). The brand of the products has an impact on customer acquisition, retention, and profitability [136]. Profitability will improve for those firms with high product innovation activity [137]. According to Bocken [138], one of the major characteristics of successful and failed sustainable businesses is the novelty of the business model. The business model includes market segments and a value proposition (*c*_41_), marketing channels, customer relations, key resources and partnerships, revenue streams and cost structure (value capture) (see [139]). Thus, our results are consistent with those found in the prior literature. 

#### 5.1.3. The Aspect of Product ($D_{5}$) and Criteria

The aspect of product ${(D}_{5}$) has been recognized as dominant for evaluating new ventures for decades. Researchers usually classify investment criteria into five classes: The personality of the entrepreneur, the experience and qualifications of the entrepreneur, the product and service, the market, and financial considerations [118]. According to Reyes-Menendez et al. [86], depending on the importance of a product or service for an individual, different factors will influence the customer’s decision. According to Fried and Hisrich [77], a business idea (new product ${(D}_{5}$), service, or retail concept) that works already or can be commercialized within two to three years (*c*_73_) should be considered by the VCs while evaluating new ventures. 

The most influential criteria from the product aspect ${(D}_{5}$) are patent protection (*c*_51_) and great growth potential (*c*_54_). The analytic result is consistent with those of other scholars. Most of the researches on the role of IP in the evaluation and selection of new ventures by VCs mainly have focused on patents; i.e., new ventures that already have patents are more likely to receive VC funds (Engel and Keilbach [140], Cao and Hsu [141], Audretsch, Bönte and Mahagaonkar [142], Häussler et al. [143]) and be valued more highly by VCs (Lerner [144], Baum and Silverman [145], Hsu and Ziedonis [146]). 

Additionally, new ventures with patents have greater performance throughout the VC cycle compared to other start-ups, in terms of both their survival possibilities and the total funds they receive [141,147]. Patent protection is especially important for medical devices firms; in particularly, firms produce spinal medical devices. According to Valoir and Paradiso [148], a lot of medical products are regulated by the FDA and cannot be commercialized without approval by FDA in advance. Likewise, a product cannot be commercialized safely if there are dominant patents that already cover that product. Therefore, both the FDA laws and the patent laws have barriers to market entry. 

For unique products and technologies (*c*_52_), according to Moini [149], a firm’s competitive pricing, technical superiority, patent holdings, and unique products can each contribute to an end product and/or a manufacturing process that is distinct. Kon et al. [150] argued that a firm’s ability to fulfill the unique requirements of customers can make a new venture successful. Based on past works (e.g., Tyebjee and Bruno [70]; Hutt and Thomas [151]; Khan [152]; Hisrich and Jankowicz [82]), numerous critical criteria which VCs use to make investment decisions have been identified. The degree of product differentiation (*c*_52_) is one of the most important of these criteria [153]. 

#### 5.1.4. The Aspect of Valuation ($D_{8}$)

A real option-based valuation ${(D}_{8}$) can be influenced by various factors. Based on the derived influence relationship being illustrated in Figure 3, that value is mainly influenced by the nature of the management team ${(D}_{1}$), professional competencies of the management team ${(D}_{2}$), and the related experience of the management team ${(D}_{3}$). These analytic results are consistent with other studies (e.g., Roberts and Weitzman [154]; McGrath [155]). 

According to McGrath and Nerkar [156], for a decision-maker using real option analysis, the value of a new option will be influenced by the presence of preexisting options. Moreover, the contribution of adopting real option analysis to a firm’s existing portfolio of R&D opportunities depends to a large extent on the firm’s prior experience ${(D}_{3}$) and the declining value of having to wait to exercise previously created options. According to Mason [157] and Bocken [138], the unjustified business models, unverified management teams ${(D}_{1}$ and $D_{3}$), novel technologies, and early markets of a new venture are all major causes of risk and uncertainty for investors [157,158]. VCs try to overcome these risks and uncertainties about possible future investments by sharing information with other investors and stakeholders. Information sharing of this type is built on mutual trust that has been earned [157]. 

#### 5.1.5. The Most Influential Criteria

According to Table 5, the most influential criteria for Aspect 1 include characters of the management team (*c*_11_) and integrity of the management team (*c*_14_). Managers’ integrity influences the capability of the firm to focus on the task constantly (*c*_15_) and the details (*c*_16_). The venture investment is characterized by high uncertainty and high levels of information asymmetry. Managers’ characters and integrity may mitigate the potential adverse selection problems. Our results are consistent with those in the prior literature. Sahlman [159] indicated that managers are likely to engage in opportunistic behavior after VCs provide funds due to severe information asymmetries. Hisrich and Jankowicz [8] indicated that VCs’ funding decisions may be affected by the personal integrity of managers. 

The most influential criteria for Aspect 2 is the ability to execute the business plan (*c*_25_). The ability of the management team to implement the business plan affects the ability to arrange R&D activity and promote new products. The result is consistent with the extant studies. MacMillan et al. [79] indicated that the venture is less likely to be mismanaged when managers can endeavor intensively. This aspect implies that the ability of management team to execute business plans can have positive impacts on a firm’s activities such as marketing or R&D. 

The actual operating performance (*c*_31_) is the most influential criterion of Aspect 3. The result is consistent with the prior research. MacMillan et al. [79] indicated that the probability of losing all investment tends to be relatively low when managers have good performance in the past. Other studies also find that VCs use the backgrounds and past successes of founders to assess the potential future success [134,160]. Managers with good actual operating performance are familiar with the market and industry as well as have related experience in evaluating risks. VCs regard a thorough familiarity with the target market as an important experience requirement. 

The most influential criterion for Aspect 4 is value proposition such as product efficacy, brand, or price (*c*_41_). Product efficacy will affect customer relations and further affect the firms’ profitability. Our result is consistent with the prior studies. Cooper [161] indicated that new products that have superior price/performance characteristics and have unique attributes for customers can be successful. Valuation proposition also affects the target market clients (*c*_42_), key resources (*c*_48_), and key activities (*c*_49_). Cooper [161] further indicated that in the early stage of developing new products, firms need to specify their target market exactly, and identify those activities having impacts on the project outcomes. 

The most influential criteria for the Aspect 5 are patent protection (*c*_51_) and great growth potential (*c*_54_). The rationality was already discussed here in the earlier Section 5.1.3. In Aspect 6, the most influential criterion is market acceptance (*c*_63_). Market acceptance influences growth potential (*c*_61_) and market demand (*c*_62_). Our results are consistent with other studies. The probability of product and market development failure will be lower when a product has demonstrated market acceptance. Thus, VCs prefer to fund those firms whose products already have clear market acceptance in an existing market [79]. The most influential criteria in Aspect 7 are shorter payback period (*c*_73_) and the withdrawal mechanisms or the investment project (*c*_75_). The rationality of these research results was already discussed in Section 5.1.1. 

### 5.2. Mutual Influences among the Most Important Aspects and Criteria

Key decision-making factors that can influence the decision of investment by VCs include the nature of the management team, the professional competencies of the management team, related experience of the management team, the business model, products, market, and the nature of the investment project. By summarizing the experts’ opinions using the modified Delphi method, the key constructs that are more dominant in the evaluation of a VC investment project are summarized in Table 3. The consistency between our empirical study results versus the past efforts will be discussed in the following sub-sections. 

#### The Influence Relationships among Constructs

Our results indicate four dominant influence relationship. First, business model ${(D}_{4}$) impacts the nature of the management team ${(D}_{1}$). Second, the product aspect ${(D}_{5}$) impacts the nature of the management team ${(D}_{1}$), professional competences of the management team ${(D}_{2}$), and the related experiences of the management team ${(D}_{3}$). Third, the market aspect ${(D}_{6}$) impacts the nature of the management team ${(D}_{1}$). Fourth, the nature of the investment project ${(D}_{7}$) impacts the nature of the management team ${(D}_{1}$), professional competences of the management team ${(D}_{2}$) and also the related experience of the management team ${(D}_{3}$). Further, in terms of construct rankings (See Table 7), the rankings of the constructs by importance are the following: the nature of the investment project ${(D}_{7}$), the business model ${(D}_{4}$), products ${(D}_{5}$), real option ${(D}_{8}$), market ${(D}_{6}$), professional competencies of the management team ${(D}_{2}$), related experience of the management team ${(D}_{3}$), and the nature of the management team ${(D}_{1}$). The consistencies between the research findings and those in past works are discussed below.

- Business Model Impacts the Nature of the Management Team

Johnson et al. [97] indicated that business models included the customer value proposition and the profit model, key resource, key process, etc. The customer value proposition and profit model define the value of customers and the organization. Key resource and key process describe how to create value for customers and the organization. The customer value proposition advocates for the value of the company to its customers through products and services and assists customers in resolving issues and fulfilling certain needs. It is believed the business model has a direct relationship with the overall nature of the management team [97].

- Products Impact the Nature of the Management Team

According to Ulrich and Eppinger [162], the organizational structure for new product development may need to align with functions, projects, or both. Further, the same firm may adopt diverse processes for different categories of the projects for the development of new products or services [162]. Project managers in general and managers of projects for new product development particularly do their jobs better and achieve better results when their characteristics and personal competences match the requirements and needs of the project [163,164]. Finally, different leadership styles need to be more appropriate to achieve project managers’ successes for different types of projects [165]. In general, different types of product development projects should influence the evaluation and selection of their managers.

- The Market Impacts the Nature of the Management Team

Ruekert [166] considers market orientation as an action that promotes employees’ turn towards market services. These actions are conducted by market information and cross-department integration and communications [166]. Auh and Menguc [167] regarded market orientation as an organizational culture that eventually creates customer value through cross-functional coordination to collect information on customers and competitors. Apparently, the market will influence the management team and organizational behavior.

- The Nature of the Investment Project Impacts the Nature of the Management Team

According to institutional theory, new ventures are very likely to duplicate both the organizational structure and activities of focal firms within their environment in order to gain legitimacy and offset the liability of newness [168,169]. Further, according to Ensley and Hmieleski [170], university-based and independent high technology start-ups may demonstrate differences in top management team composition, dynamics and performance. Apparently, the nature of the investment project impacts the nature of the management team. The analytic results are consistent with past works.

- Potential Growth of the Market Impacts the Nature of the Investment Project

Tyebjee and Bruno [70] indicated that one of five key points during the process of deal evaluation of an investment project is market attractiveness. The growth potential of the market is included as part of market attractiveness. Amitet al. [97] believed the key points for VCs to evaluate an investment project include market potential, specialization in a specific industry, and a positive position at a particular investment stage. Apparently, market attractiveness and growth potential influence VCs’ investment decisions, and thus the nature of the investment project.

- Market Demands and Market Acceptance Impact the Nature of the Investment Project

Hall and Hofer [83] indicated that one of the key factors for VCs to use to evaluate an investment project is market characteristics and size of the products. Market demand is included within market characteristics and size of the product or products [83]. Thus, market demand impacts the nature of the investment project. Market acceptance is also included within market characteristics and size of the product/s [83]. Thus, market acceptance impacts the nature of the investment project. The influence relationship being derived is consistent with the work by Hall and Hofer [83].

### 5.3. Advances in Research Methods

In this work, a novel analytical framework consisting of the DANP-mV and ROV methods was proposed. The proposed analytical framework can overcome the limitations of the traditional ROV methods, which do not consider qualitative and local criteria or factors. The integrated model also overcomes the limitations of the MCDM framework. Further, the adoption of DANP can raise the assumptions of the independence between aspects and criteria. The introduction of the modified VIKOR method can overcome the restrictions of the TOPSIS method uncovered by [91]. Further, the target levels of the criteria were considered by the modified VIKOR method. In this work, the proposed analytical framework was applied in evaluating spinal medical device firms. In the future, the well-verified analytical framework can be applied to investment projects in other industries.

## 6. Implications

### 6.1. Managerial Implications

According to the empirical study results, the business model, nature of the investment, product, and value of the investment are the most dominant aspects. These aspects can be used to evaluate and select an appropriate spinal medical device firm for investment. To prevent the possibility of picking the best apple from a barrel of rotten apples, after the evaluation and selection of the target firm for investment, the selected spinal medical device firm should be enhanced further through the most influential aspects. Based on the analytical results derived by DEMATEL, the professional competencies of the management team and the nature of the management team were the most influential ones. Thus, the target firm can further be enhanced by strengthening the professional competencies of the management team and improving the nature of the management team. Then, the target level of professional competencies and the nature of the management team can be enhanced further to achieve the target level.

### 6.2. Practical Implications

It is important for VCs to evaluate a potential investment that can produce high returns while minimizing risk. Traditionally, VCs use DCF methods to evaluate the feasibility of investment projects. We propose that VCs use the real option- and DANP-mV-based MCDM method, which takes different levels of risk and market conditions into consideration. VCs can use the integrated method to choose a promising investment project more accurately than they could do using DCF. In addition, VCs can focus on the most influential criteria and the mutual impacts among different criteria to evaluate the investment project by using the integrated method. We suggest that the most important criteria for VCs to choose a good investment project include the character of the management team, integrity of the management team, the capacity to execute the business plans, operating performance, value proposition, patent protection, great growth potential, and market acceptance. We also suggest that VCs need to consider the mutual impacts. The nature of the management team affects the quality of business models, new product development, and the nature of the investment project. The nature of the investment project is affected by potential market growth, market demands, and market acceptance.

## 7. Limitations and Future Research Possibilities

This study has several limitations. First is the accuracy of the financial statement. The S company in this study is a private startup that is not listed on the Taiwan Stock Exchange or Over-the-Counter market in Taiwan. Due to the nature of the company, its research and development information is also considered to be a trade secret. Thus, we were unable to obtain the company’s public information. The financial data of the S company were forecasted by experts based on information provided by the S company. This study eliminates any preventable variables and searches for as much related and similar public information from similar industries as possible in order to obtain the most accurate information. However, the end result of this study might still be compromised.

Second are the systematic errors and risks of the real option method. Taking the real application into consideration, this study situates the S company so as to face four different strategic scenarios: defer, abandon, contract, and expand. However, S company might face more than four types of scenarios after it puts its investment project into practice. Further, this study does not consider the possible interference among four offered scenarios. The research method of this study used the evaluation mode of a bi-normal option with redefined variables. Due to the simple scenarios and assumptions in this study, it is believed that the results might be different from reality.

For any future research possibilities, using both actual historical financial data and forecasted financial information of investment projects will definitely help VCs make better judgments on the future development potential of a company, have better control over the environmental changes within the industry, and make more realistic assessments of the outcomes with a broader range. Furthermore, besides newly established medical device companies, the research methods of this study can also be applied to other industries. It is believed, therefore, that this study will help VCs achieve better judgments of their future investment evaluations.

## 8. Conclusions

Spinal surgical device firms with great growth potentials have become the investment targets of VCs, who always focus on high-growth industries. It is important for VCs to evaluate a potential investment that can produce big returns while minimizing risk. Traditionally, VCs use DCF methods such as NPV or IRR to evaluate the feasibility of investment projects. We propose that VCs use the real option- and DANP-mV-based MCDM method, which takes different levels of risk and market conditions into consideration. By using the proposed real option analysis-based MCDM framework, VCs can more precisely evaluate these investment proposals. Accurate valuation results will not only help provide important information on strategic decision-making for the CEO but also help VCs obtain an accurate value of an investment proposal. Through the integrated method, VCs can make correct decisions about newly established spinal medical device firms.

## Figures and Tables

**Table 1:** Discounted Cash Flow Evaluation Methods: The pros and cons.

Evaluation Method	Principle	Pros	Cons
NPV	NPV > 0. Working with IRR becomes the major strategic decision-making method.	Takes the time value of money and cash flow into consideration.	The NPV method does not give visibility for how long a project will take to generate a positive NPV.
IRR	IRR > opportunity cost of capital. Working with NPV has become the major strategic decision-making method.	Takes both the time value of money and cash flow into consideration.	There may be no or multiple results.

Source: Summarized by this research effort. NPV—net present value; IRR— internal rate of return.

**Table 2:** Categories of Real Options.

Categories	Description	Applicable Industry	References
Option to Defer	Obtain a lease or call for an object that can be leased or bought under the promised condition in the agreed period of time.	Natural resource extraction industry, Land development industry, agricultural industry, paper industry, etc.	McDonald and Siegel [43], Paddock et al. [44], Tourinho [45], Titman [46], Ingersoll and Ross [47]
Time-to-Build Option	The result of the time-to-build option will affect the execution of the next stage. This is a compound option.	Pharmaceutical industry, start-up high tech industry, large-scale public construction, or power plant construction.	Majd and Pindyck [48], Carr [49], Trigeorgis [50]
Option to Expand	Expand the scale of production or accelerate resource utilization if market conditions become more favorable than expected.	Natural resources industry, seasonal facilities planning and construction, garment industry, consumption property industry, etc.	Trigeorgis and Mason [51], Pindyck [52], McDonald and Seigel [53,54]
Option to Contract	Reduce the scale of production or lower resource utilization if market conditions become less favorable than expected	Natural resources industry, seasonal facilities planning and construction, garment industry, consumption property industry, etc.	Trigeorgis and Mason [51], Pindyck [52], McDonald and Seigel [53,54]
Option to Shut Down and Restart Operations	Determine if the project should be shut down or restarted based on the market situation.	Natural resources industry, seasonal facilities planning and construction, garment industry, consumption property industry, etc.	Trigeorgis and Mason [51], Pindyck [52], McDonald and Seigel [53,54]
Option to Abandon	Abandon the investment project to make up for the loss of assets.	Transportation industry, investment and financial services, introduction of new products into an uncertain market	Myers and Majd [55]
Option to Switch	According to the actual situation, adjust or change the strategy for the investment project.	Power industry, chemical industry, raw material industry, components industry	Margrabe [56], Kensinger [57], Kulatilaka and Trigeorgis [58]
Growth Options	To increase the growth of the investment project in the future, it will need to execute other investment projects to obtain growth of the option value	Infrastructure industry, high-tech industry, complex or multi-nationality business operation, strategic merger and acquisition, etc.	Myers [8], Kester [59,60], Trigeorgis [61], Chung and Chjaroenwong [62]
Multiple Interacting Options	Obtain multiple options that will interact and affect each other. It is also called the portfolio strategic option.	This method can be applied to all industry.	Trigeorgis [63], Brennan and Schwartz [54], Kulatilaka and Trigeorgis [64]

Source: Trigeorgis [42].

**Table 3:** Criteria Definitions and Reference.

Construct	Criteria	Reference
The nature of the management team (*D*_1_)	Characters of the management team (*c*_11_)	Tyebjee and Bruno [70], Davis and Stetson [95], MacMillanet al. [79], Amit, Glosten and Muller [96]
	Business philosophy of the management team (*c*_12_)	Davis and Stetson [95], MacMillanet al. [79]
	Leadership of the management team (*c*_13_)	MacMillanet al. [79], Fried and Hisrich [77]
	Integrity of the management team (*c*_14_)	Fried and Hisrich [77]
	The capability to focus on the tasks constantly (*c*_15_)	MacMillanet al. [79]
	The capability to focus on details (*c*_16_)	MacMillanet al. [79]
Professional competences of the management team (*D*_2_)	Financial ability of the management team (*c*_21_)	Tyebjee and Bruno [70], MacMillanet al. [79]
	Marketing ability of the management team (*c*_22_)	Tyebjee and Bruno [70], MacMillanet al. [79]
	R&D ability of the management team (*c*_23_)	Tyebjee and Bruno [70], MacMillanet al. [79]
	Productivity of the management team (*c*_24_)	MacMillanet al. [79]
	The ability of executing the business plan (*c*_25_)	MacMillanet al. [79]
Related experience of the management team (*D*_3_)	The actual operating performance (*c*_31_)	Davis and Stetson [95], MacMillanet al. [79], Fried and Hisrich [77]
	Familiarity of the market and industry (*c*_32_)	Davis and Stetson [95], MacMillanet al. [79], Hall and Hofer [83], Fried and Hisrich [77]
	The coping abilities of the economy-cycle (*c*_33_)	Tyebjee and Bruno [70]
	The ability to evaluate and respond to potential risks (*c*_34_)	Tyebjee and Bruno [70], MacMillanet al. [79]
	The ability to demonstrate products and operations (*c*_35_)	MacMillanet al. [79]
	The ability to prevent R&D degradation (*c*_36_)	Tyebjee and Bruno [70]
Business model (*D*_4_)	Value proposition (product efficacy, brand, price) (*c*_41_)	Johnson, Christensen and Kagermann [97], Osterwalder and Pigneur [98]
	Target market clients (*c*_42_)	Johnsonet al. [97], Osterwalder and Pigneur [98]
	Sales channel (*c*_43_)	Johnsonet al. [97], Osterwalder and Pigneur [98]
	Customer relation (*c*_44_)	Osterwalder and Pigneur [98]
	Key partners (*c*_45_)	Johnsonet al. [97], Osterwalder and Pigneur [98]
	The main source of profit (*c*_46_)	Johnsonet al. [97], Osterwalder and Pigneur [98]
	Cost structure (*c*_47_)	Johnsonet al. [97], Osterwalder and Pigneur [98]
	Key resources (*c*_48_)	Osterwalder and Pigneur [98]
	Key activities (*c*_40_)	Osterwalder and Pigneur [98]
Products (*D*_5_)	Patent protection (*c*_51_)	Tyebjee and Bruno [70], MacMillanet al. [79]
	Unique products and technologies (*c*_52_)	Tyebjee and Bruno [70], Amitet al. [96]
	Competitors entry barriers (*c*_53_)	Tyebjee and Bruno [70], MacMillanet al. [79]
	Great growth potential of operating profit (*c*_54_)	Amitet al. [96], Fried and Hisrich [77]
	Technical level of the products (*c*_55_)	Tyebjee and Bruno [70]
	Gross profit of the products (*c*_56_)	Tyebjee and Bruno [70]
Market (*D*_6_)	Growth potential (*c*_61_)	Tyebjee and Bruno [70], MacMillanet al. [79], Amitet al. [96], Fried and Hisrich [77]
	Market demands (*c*_62_)	Tyebjee and Bruno [70], MacMillanet al. [79], Hall and Hofer [83]
	Market acceptance (*c*_63_)	Tyebjee and Bruno [70], MacMillanet al. [79]
The nature of the investment project (*D*_7_)	Investment scale and strategies (*c*_71_)	Davis and Stetson (1985), Amitet al. [96], Hall and Hofer [83]
	The estimated ROI ratio of the investment projects (*c*_72_)	MacMillanet al. [79], Hall and Hofer [83], Fried and Hisrich [77]
	Shorter recall period (about 2~3 years) (*c*_73_)	Tyebjee and Bruno [70], MacMillanet al. [79], Fried and Hisrich [77]
	The potential of being merged and acquired (*c*_74_)	Tyebjee and Bruno [70]
	The withdrawal mechanisms of the investment project (*c*_75_)	Tyebjee and Bruno [70], Davis and Stetson [95], MacMillanet al. [79], Hall and Hofer [83], Fried and Hisrich [77]
Value of the investment (*D*_8_)	Value of real option	Myers [8], Kodukula and Papudesu [99], Schulmerich [9]

Source. This study.

**Table 4:** Experts’ Background.

No.	Education	Title	Experience (year)
1	Ph.D.	Associate	9
2	Master	Vice President	26
3	Master	Vice President	10
4	Master	Associate	11
5	Master	Manager	12
6	Master	Manager	12
7	Master	Manager	8
8	Master	Deputy Manager	8
9	Bachelor	Deputy Manager	10

**Table 5:** The values of $r_{i},\;{\;c}_{i},\;r_{i}{+c}_{i},\;r_{i}{-c}_{i}$ and weight versus each Aspect and Criterion.

**Aspect**	***r***	***c***	***r* + *c***	***r* − *c***	**Weight**
*D*_1_	12.564	10.772	23.336	1.791	11.636%
*D*_2_	12.577	11.157	23.733	1.420	12.052%
*D*_3_	12.095	11.093	23.188	1.002	11.986%
*D*_4_	11.322	12.098	23.420	−0.776	13.081%
*D*_5_	11.038	12.012	23.050	−0.974	12.990%
*D*_6_	11.463	11.551	23.014	−0.088	12.488%
*D*_7_	10.819	12.145	22.963	−1.326	13.135%
*D*_8_	10.631	11.679	22.310	−1.048	12.632%
**Criteria**	***r***	***c***	***r* + *c***	***r* − *c***	**Weight**
*c*_11_	17.524	16.075	33.600	1.449	2.027%
*c*_12_	16.582	16.713	33.295	−0.131	2.030%
*c*_13_	16.648	16.432	33.079	0.216	2.029%
*c*_14_	17.310	16.065	33.375	1.245	2.027%
*c*_15_	15.700	17.014	32.714	−1.313	2.031%
*c*_16_	15.191	16.657	31.848	−1.466	2.030%
*c*_21_	9.979	9.915	19.894	0.065	2.162%
*c*_22_	9.670	9.698	19.367	−0.028	2.160%
*c*_23_	9.362	9.864	19.226	−0.502	2.161%
*c*_24_	9.373	9.865	19.238	−0.492	2.161%
*c*_25_	10.870	9.914	20.784	0.956	2.162%
*c*_31_	17.676	15.724	33.400	1.952	2.097%
*c*_32_	16.691	17.218	33.909	−0.526	2.104%
*c*_33_	16.401	17.082	33.483	−0.681	2.104%
*c*_34_	16.860	17.301	34.161	−0.441	2.105%
*c*_35_	15.927	15.923	31.850	0.005	2.098%
*c*_36_	16.317	16.624	32.941	-0.307	2.101%
*c*_41_	19.034	18.105	37.139	0.929	2.156%
*c*_42_	18.475	18.208	36.683	0.267	2.157%
*c*_43_	18.264	17.950	36.214	0.314	2.155%
**Criteria**	***r***	***c***	***r* + *c***	***r* − *c***	**Weight**
*c*_44_	17.649	17.897	35.546	−0.248	2.155%
*c*_45_	17.732	17.739	35.471	−0.006	2.153%
*c*_46_	18.152	18.569	36.721	−0.416	2.160%
*c*_47_	17.690	17.795	35.485	−0.105	2.154%
*c*_48_	17.675	17.842	35.517	−0.167	2.154%
*c*_49_	17.485	18.051	35.536	−0.566	2.156%
*c*_51_	17.524	16.075	33.600	1.449	2.278%
*c*_52_	16.582	16.713	33.295	−0.131	2.281%
*c*_53_	16.648	16.432	33.079	0.216	2.282%
*c*_54_	17.310	16.065	33.375	1.245	2.281%
*c*_55_	15.700	17.014	32.714	−1.313	2.281%
*c*_56_	15.191	16.657	31.848	−1.466	2.284%
*c*_61_	61.825	62.846	124.671	−1.021	2.346%
*c*_62_	60.770	62.327	123.097	−1.557	2.346%
*c*_63_	62.846	60.268	123.114	2.578	2.344%
*c*_71_	25.424	26.952	52.376	−1.528	2.334%
*c*_72_	25.713	27.221	52.934	−1.508	2.335%
*c*_73_	26.520	25.581	52.101	0.938	2.330%
*c*_74_	26.111	24.411	50.522	1.700	2.326%
*c*_75_	27.233	26.835	54.068	0.398	2.333%

**Table 6:** Normalized real option valuation (ROV) results for three companies.

Investment Projects	S	W	B
Normalized Results	5.000	4.527	4.036

**Table 7:** Performance Scores of the Three Investment Projects.

Criteria	S	W	B	Criteria	S	W	B
*c*_11_	4.556	4.333	3.889	*c*_46_	4.444	3.889	4.000
*c*_12_	4.667	4.111	3.889	*c*_47_	4.778	3.889	3.556
*c*_13_	4.778	4.111	3.667	*c*_48_	4.778	3.889	3.889
*c*_14_	4.444	4.222	3.444	*c*_49_	5.000	4.222	3.778
*c*_15_	4.889	4.444	3.778	*c*_51_	4.222	3.889	3.889
*c*_16_	4.111	4.000	3.333	*c*_52_	4.333	4.000	3.778
*c*_21_	4.667	4.111	3.667	*c*_53_	5.000	4.667	4.000
*c*_22_	4.667	3.889	4.000	*c*_54_	4.556	4.000	4.000
*c*_23_	4.778	4.222	4.111	*c*_55_	4.667	4.667	4.111
*c*_24_	4.444	4.111	3.556	*c*_56_	4.889	4.222	3.889
*c*_25_	4.444	4.556	3.667	*c*_61_	4.667	4.111	4.000
*c*_31_	4.333	4.000	4.000	*c*_62_	4.556	4.667	4.111
*c*_32_	4.333	4.000	3.667	*c*_63_	5.000	4.222	3.889
*c*_33_	4.778	4.667	4.000	*c*_71_	4.778	4.222	3.556
*c*_34_	5.000	3.889	3.889	*c*_72_	4.222	4.000	3.889
*c*_35_	4.556	4.333	3.778	*c*_73_	4.889	4.333	3.889
*c*_36_	4.889	4.111	3.889	*c*_74_	4.667	4.333	3.778
*c*_41_	5.000	5.000	3.667	*c*_75_	4.444	4.111	3.778
*c*_42_	4.111	4.222	3.778	*D*_8_	5.000	4.527	4.036
*c*_43_	4.889	4.333	3.556				
*c*_44_	4.111	4.000	3.556	Vikor	0.000	0.494	1.000
*c*_45_	4.667	4.000	3.444	Rank	1	2	3

## Appendix A. Results of Modified Delphi Method

**Table A1:** Results being derived by Using the Modified Delphi Method.

No.			D_1_						D_2_					D_3_								D_4_							D_5_					D_6_				D_7_			D8
	*c*_8_	*c*_14_	*c*_15_	*c*_24_	*c*_25_	*c*_28_	*c*_10_	*c*_11_	*c*_12_	*c*_13_	*c*_16_	*c*_9_	*c*_17_	*c*_18_	*c*_26_	*c*_27_	*c*_29_	*c*_31_	*c*_32_	*c*_33_	*c*_34_	*c*_35_	*c*_36_	*c*_37_	*c*_39_	*c*_40_	*c*_1_	*c*_5_	*c*_6_	*c*_7_	*c*_23_	*c*_38_	*c*_2_	*c*_3_	*c*_4_	*c*_19_	*c*_20_	*c*_21_	*c*_22_	*c*_30_	-
1	A	A	A	A	A	A	A	A	A	A	A	A	A	A	A	A	A	A	A	A	A	A	A	A	A	A	A	A	A	A	A	A	A	A	A	A	A	A	A	A	A
2	A	A	A	A	A	A	A	A	A	A	A	A	A	A	A	A	A	A	A	A	A	A	A	A	A	A	A	A	A	A	A	A	A	A	A	A	A	A	A	A	A
3	A	A	A	A	A	A	A	A	A	A	A	A	A	A	A	A	A	A	A	A	A	A	A	A	A	A	A	A	A	A	A	A	A	A	A	A	A	A	A	A	A
4	A	A	A	A	A	A	A	A	A	A	A	A	A	A	A	A	A	A	A	A	A	A	A	A	A	A	A	A	A	A	A	A	A	A	A	A	A	A	A	A	A
5	A	A	A	A	A	A	A	A	A	A	A	A	A	A	A	A	A	A	A	A	A	A	A	A	A	A	A	A	A	A	A	A	A	A	A	A	A	A	A	A	A
6	A	A	A	A	A	A	A	A	A	A	A	A	A	A	A	A	A	A	A	A	A	A	A	A	A	A	A	A	A	A	A	A	A	A	A	A	A	A	A	A	A
7	A	A	A	A	A	A	A	A	A	A	A	A	A	A	A	A	A	A	A	A	A	A	A	A	A	A	A	A	A	A	A	A	A	A	A	A	A	A	A	A	A
8	A	A	A	A	A	A	A	A	A	A	A	A	A	A	A	A	A	A	A	A	A	A	A	A	A	A	A	A	A	A	A	A	A	A	A	A	A	A	A	A	A
9	A	A	A	A	A	A	A	A	A	A	A	A	A	A	A	A	A	A	A	A	A	A	A	A	A	A	A	A	A	A	A	A	A	A	A	A	A	A	A	A	A
Agree	9	9	9	9	9	9	9	9	9	9	9	9	9	9	9	9	9	9	9	9	9	9	9	9	9	9	9	9	9	9	9	9	9	9	9	9	9	9	9	9	9
(%)	100%																																								

## Appendix C. The Normalized and Total Relationship Matrices

**Table A2:** Normalized Matrix.

***N***=			D1-1	D1-2	D1-3	D1-4	D1-5	D1-6	D2-1	D2-2	D2-3	D2-4	D2-5	D3-1	D3-2	D3-3	D3-4	D3-5	D3-6	D4-1	D4-2	D4-3	D4-4	D4-5	D4-6	D4-7	D4-8	D4-9	D5-1	D5-2	D5-3	D5-4	D5-5	D5-6	D6-1	D6-2	D6-3	D7-1	D7-2	D7-3	D7-4	D7-5	
	D1-1		0.000	0.006	0.006	0.006	0.006	0.006	0.031	0.031	0.031	0.031	0.031	0.029	0.029	0.029	0.029	0.029	0.029	0.028	0.028	0.028	0.028	0.028	0.028	0.028	0.028	0.028	0.028	0.028	0.028	0.028	0.028	0.028	0.026	0.026	0.026	0.028	0.028	0.028	0.028	0.028	
	D1-2		0.006	0.000	0.005	0.006	0.006	0.006	0.029	0.029	0.029	0.029	0.029	0.027	0.027	0.027	0.027	0.027	0.027	0.026	0.026	0.026	0.026	0.026	0.026	0.026	0.026	0.026	0.026	0.026	0.026	0.026	0.026	0.026	0.025	0.025	0.025	0.026	0.026	0.026	0.026	0.026	
	D1-3		0.006	0.006	0.000	0.006	0.006	0.006	0.029	0.029	0.029	0.029	0.029	0.027	0.027	0.027	0.027	0.027	0.027	0.027	0.027	0.027	0.027	0.027	0.027	0.027	0.027	0.027	0.027	0.027	0.027	0.027	0.027	0.027	0.025	0.025	0.025	0.027	0.027	0.027	0.027	0.027	
	D1-4		0.006	0.006	0.006	0.000	0.006	0.006	0.030	0.030	0.030	0.030	0.030	0.028	0.028	0.028	0.028	0.028	0.028	0.027	0.027	0.027	0.027	0.027	0.027	0.027	0.027	0.027	0.027	0.027	0.027	0.027	0.027	0.027	0.026	0.026	0.026	0.027	0.027	0.027	0.027	0.027	
	D1-5		0.005	0.006	0.006	0.005	0.000	0.006	0.028	0.028	0.028	0.028	0.028	0.026	0.026	0.026	0.026	0.026	0.026	0.025	0.025	0.025	0.025	0.025	0.025	0.025	0.025	0.025	0.025	0.025	0.025	0.025	0.025	0.025	0.024	0.024	0.024	0.025	0.025	0.025	0.025	0.025	
	D1-6		0.005	0.005	0.005	0.005	0.006	0.000	0.027	0.027	0.027	0.027	0.027	0.025	0.025	0.025	0.025	0.025	0.025	0.024	0.024	0.024	0.024	0.024	0.024	0.024	0.024	0.024	0.024	0.024	0.024	0.024	0.024	0.024	0.023	0.023	0.023	0.024	0.024	0.024	0.024	0.024	
	D2-1		0.026	0.026	0.026	0.026	0.026	0.026	0.000	0.005	0.005	0.005	0.006	0.025	0.025	0.025	0.025	0.025	0.025	0.025	0.025	0.025	0.025	0.025	0.025	0.025	0.025	0.025	0.027	0.027	0.027	0.027	0.027	0.027	0.025	0.025	0.025	0.027	0.027	0.027	0.027	0.027	
	D2-2		0.025	0.025	0.025	0.025	0.025	0.025	0.006	0.000	0.005	0.005	0.006	0.024	0.024	0.024	0.024	0.024	0.024	0.024	0.024	0.024	0.024	0.024	0.024	0.024	0.024	0.024	0.026	0.026	0.026	0.026	0.026	0.026	0.024	0.024	0.024	0.026	0.026	0.026	0.026	0.026	
	D2-3		0.025	0.025	0.025	0.025	0.025	0.025	0.005	0.005	0.000	0.005	0.005	0.023	0.023	0.023	0.023	0.023	0.023	0.023	0.023	0.023	0.023	0.023	0.023	0.023	0.023	0.023	0.025	0.025	0.025	0.025	0.025	0.025	0.023	0.023	0.023	0.025	0.025	0.025	0.025	0.025	
	D2-4		0.025	0.025	0.025	0.025	0.025	0.025	0.005	0.005	0.005	0.000	0.005	0.023	0.023	0.023	0.023	0.023	0.023	0.023	0.023	0.023	0.023	0.023	0.023	0.023	0.023	0.023	0.025	0.025	0.025	0.025	0.025	0.025	0.023	0.023	0.023	0.025	0.025	0.025	0.025	0.025	
	D2-5		0.028	0.028	0.028	0.028	0.028	0.028	0.006	0.006	0.006	0.006	0.000	0.027	0.027	0.027	0.027	0.027	0.027	0.027	0.027	0.027	0.027	0.027	0.027	0.027	0.027	0.027	0.029	0.029	0.029	0.029	0.029	0.029	0.027	0.027	0.027	0.029	0.029	0.029	0.029	0.029	
	D3-1		0.026	0.026	0.026	0.026	0.026	0.026	0.028	0.028	0.028	0.028	0.028	0.000	0.006	0.006	0.006	0.006	0.006	0.027	0.027	0.027	0.027	0.027	0.027	0.027	0.027	0.027	0.027	0.027	0.027	0.027	0.027	0.027	0.025	0.025	0.025	0.024	0.024	0.024	0.024	0.024	
	D3-2		0.025	0.025	0.025	0.025	0.025	0.025	0.026	0.026	0.026	0.026	0.026	0.005	0.000	0.006	0.006	0.006	0.006	0.025	0.025	0.025	0.025	0.025	0.025	0.025	0.025	0.025	0.025	0.025	0.025	0.025	0.025	0.025	0.023	0.023	0.023	0.023	0.023	0.023	0.023	0.023	
	D3-3		0.024	0.024	0.024	0.024	0.024	0.024	0.026	0.026	0.026	0.026	0.026	0.005	0.006	0.000	0.006	0.005	0.005	0.025	0.025	0.025	0.025	0.025	0.025	0.025	0.025	0.025	0.025	0.025	0.025	0.025	0.025	0.025	0.023	0.023	0.023	0.022	0.022	0.022	0.022	0.022	
	D3-4		0.025	0.025	0.025	0.025	0.025	0.025	0.026	0.026	0.026	0.026	0.026	0.006	0.006	0.006	0.000	0.005	0.006	0.026	0.026	0.026	0.026	0.026	0.026	0.026	0.026	0.026	0.026	0.026	0.026	0.026	0.026	0.026	0.024	0.024	0.024	0.023	0.023	0.023	0.023	0.023	
	D3-5		0.024	0.024	0.024	0.024	0.024	0.024	0.025	0.025	0.025	0.025	0.025	0.005	0.006	0.005	0.005	0.000	0.005	0.024	0.024	0.024	0.024	0.024	0.024	0.024	0.024	0.024	0.024	0.024	0.024	0.024	0.024	0.024	0.022	0.022	0.022	0.022	0.022	0.022	0.022	0.022	
	D3-6		0.024	0.024	0.024	0.024	0.024	0.024	0.025	0.025	0.025	0.025	0.025	0.005	0.006	0.006	0.006	0.005	0.000	0.025	0.025	0.025	0.025	0.025	0.025	0.025	0.025	0.025	0.025	0.025	0.025	0.025	0.025	0.025	0.023	0.023	0.023	0.022	0.022	0.022	0.022	0.022	
	D4-1		0.022	0.022	0.022	0.022	0.022	0.022	0.023	0.023	0.023	0.023	0.023	0.023	0.023	0.023	0.023	0.023	0.023	0.000	0.006	0.006	0.006	0.006	0.006	0.006	0.006	0.006	0.026	0.026	0.026	0.026	0.026	0.026	0.026	0.026	0.026	0.027	0.027	0.027	0.027	0.027	
	D4-2		0.021	0.021	0.021	0.021	0.021	0.021	0.022	0.022	0.022	0.022	0.022	0.022	0.022	0.022	0.022	0.022	0.022	0.006	0.000	0.006	0.006	0.006	0.006	0.006	0.006	0.006	0.025	0.025	0.025	0.025	0.025	0.025	0.025	0.025	0.025	0.026	0.026	0.026	0.026	0.026	
	D4-3		0.021	0.021	0.021	0.021	0.021	0.021	0.022	0.022	0.022	0.022	0.022	0.022	0.022	0.022	0.022	0.022	0.022	0.006	0.006	0.000	0.006	0.006	0.006	0.005	0.006	0.006	0.025	0.025	0.025	0.025	0.025	0.025	0.025	0.025	0.025	0.026	0.026	0.026	0.026	0.026	
	D4-4		0.020	0.020	0.020	0.020	0.020	0.020	0.021	0.021	0.021	0.021	0.021	0.021	0.021	0.021	0.021	0.021	0.021	0.006	0.006	0.006	0.000	0.005	0.006	0.006	0.005	0.005	0.024	0.024	0.024	0.024	0.024	0.024	0.024	0.024	0.024	0.025	0.025	0.025	0.025	0.025	
	D4-5		0.021	0.021	0.021	0.021	0.021	0.021	0.021	0.021	0.021	0.021	0.021	0.021	0.021	0.021	0.021	0.021	0.021	0.006	0.006	0.005	0.005	0.000	0.006	0.006	0.006	0.006	0.024	0.024	0.024	0.024	0.024	0.024	0.024	0.024	0.024	0.025	0.025	0.025	0.025	0.025	
	D4-6		0.021	0.021	0.021	0.021	0.021	0.021	0.022	0.022	0.022	0.022	0.022	0.022	0.022	0.022	0.022	0.022	0.022	0.006	0.006	0.006	0.006	0.006	0.000	0.006	0.006	0.006	0.025	0.025	0.025	0.025	0.025	0.025	0.025	0.025	0.025	0.026	0.026	0.026	0.026	0.026	
	D4-7		0.021	0.021	0.021	0.021	0.021	0.021	0.021	0.021	0.021	0.021	0.021	0.021	0.021	0.021	0.021	0.021	0.021	0.006	0.006	0.006	0.005	0.006	0.006	0.000	0.006	0.006	0.024	0.024	0.024	0.024	0.024	0.024	0.024	0.024	0.024	0.025	0.025	0.025	0.025	0.025	
	D4-8		0.021	0.021	0.021	0.021	0.021	0.021	0.021	0.021	0.021	0.021	0.021	0.021	0.021	0.021	0.021	0.021	0.021	0.005	0.005	0.005	0.006	0.006	0.006	0.006	0.000	0.006	0.024	0.024	0.024	0.024	0.024	0.024	0.024	0.024	0.024	0.025	0.025	0.025	0.025	0.025	
	D4-9		0.020	0.020	0.020	0.020	0.020	0.020	0.021	0.021	0.021	0.021	0.021	0.021	0.021	0.021	0.021	0.021	0.021	0.006	0.006	0.006	0.006	0.006	0.006	0.005	0.006	0.000	0.024	0.024	0.024	0.024	0.024	0.024	0.024	0.024	0.024	0.025	0.025	0.025	0.025	0.025	
	D5-1		0.020	0.020	0.020	0.020	0.020	0.020	0.021	0.021	0.021	0.021	0.021	0.023	0.023	0.023	0.023	0.023	0.023	0.026	0.026	0.026	0.026	0.026	0.026	0.026	0.026	0.026	0.000	0.006	0.006	0.006	0.006	0.006	0.027	0.027	0.027	0.025	0.025	0.025	0.025	0.025	
	D5-2		0.019	0.019	0.019	0.019	0.019	0.019	0.021	0.021	0.021	0.021	0.021	0.022	0.022	0.022	0.022	0.022	0.022	0.025	0.025	0.025	0.025	0.025	0.025	0.025	0.025	0.025	0.006	0.000	0.006	0.006	0.006	0.006	0.026	0.026	0.026	0.025	0.025	0.025	0.025	0.025	
	D5-3		0.019	0.019	0.019	0.019	0.019	0.019	0.021	0.021	0.021	0.021	0.021	0.022	0.022	0.022	0.022	0.022	0.022	0.025	0.025	0.025	0.025	0.025	0.025	0.025	0.025	0.025	0.006	0.006	0.000	0.006	0.006	0.006	0.026	0.026	0.026	0.025	0.025	0.025	0.025	0.025	
	D5-4		0.018	0.018	0.018	0.018	0.018	0.018	0.019	0.019	0.019	0.019	0.019	0.020	0.020	0.020	0.020	0.020	0.020	0.023	0.023	0.023	0.023	0.023	0.023	0.023	0.023	0.023	0.005	0.005	0.005	0.000	0.005	0.006	0.024	0.024	0.024	0.023	0.023	0.023	0.023	0.023	
	D5-5		0.020	0.020	0.020	0.020	0.020	0.020	0.021	0.021	0.021	0.021	0.021	0.022	0.022	0.022	0.022	0.022	0.022	0.026	0.026	0.026	0.026	0.026	0.026	0.026	0.026	0.026	0.006	0.006	0.006	0.006	0.000	0.006	0.026	0.026	0.026	0.025	0.025	0.025	0.025	0.025	
	D5-6		0.018	0.018	0.018	0.018	0.018	0.018	0.019	0.019	0.019	0.019	0.019	0.020	0.020	0.020	0.020	0.020	0.020	0.023	0.023	0.023	0.023	0.023	0.023	0.023	0.023	0.023	0.005	0.005	0.005	0.006	0.005	0.000	0.024	0.024	0.024	0.023	0.023	0.023	0.023	0.023	
	D6-1		0.021	0.021	0.021	0.021	0.021	0.021	0.022	0.022	0.022	0.022	0.022	0.022	0.022	0.022	0.022	0.022	0.022	0.029	0.029	0.029	0.029	0.029	0.029	0.029	0.029	0.029	0.028	0.028	0.028	0.028	0.028	0.028	0.000	0.006	0.006	0.028	0.028	0.028	0.028	0.028	
	D6-2		0.021	0.021	0.021	0.021	0.021	0.021	0.022	0.022	0.022	0.022	0.022	0.022	0.022	0.022	0.022	0.022	0.022	0.029	0.029	0.029	0.029	0.029	0.029	0.029	0.029	0.029	0.027	0.027	0.027	0.027	0.027	0.027	0.006	0.000	0.006	0.027	0.027	0.027	0.027	0.027	
	D6-3		0.022	0.022	0.022	0.022	0.022	0.022	0.023	0.023	0.023	0.023	0.023	0.023	0.023	0.023	0.023	0.023	0.023	0.030	0.030	0.030	0.030	0.030	0.030	0.030	0.030	0.030	0.028	0.028	0.028	0.028	0.028	0.028	0.006	0.006	0.000	0.028	0.028	0.028	0.028	0.028	
	D7-1		0.020	0.020	0.020	0.020	0.020	0.020	0.020	0.020	0.020	0.020	0.020	0.021	0.021	0.021	0.021	0.021	0.021	0.023	0.023	0.023	0.023	0.023	0.023	0.023	0.023	0.023	0.021	0.021	0.021	0.021	0.021	0.021	0.021	0.021	0.021	0.000	0.006	0.005	0.005	0.006	
	D7-2		0.020	0.020	0.020	0.020	0.020	0.020	0.020	0.020	0.020	0.020	0.020	0.021	0.021	0.021	0.021	0.021	0.021	0.023	0.023	0.023	0.023	0.023	0.023	0.023	0.023	0.023	0.022	0.022	0.022	0.022	0.022	0.022	0.021	0.021	0.021	0.006	0.000	0.005	0.005	0.006	
	D7-3		0.021	0.021	0.021	0.021	0.021	0.021	0.021	0.021	0.021	0.021	0.021	0.022	0.022	0.022	0.022	0.022	0.022	0.024	0.024	0.024	0.024	0.024	0.024	0.024	0.024	0.024	0.022	0.022	0.022	0.022	0.022	0.022	0.022	0.022	0.022	0.006	0.006	0.000	0.005	0.006	
	D7-4		0.021	0.021	0.021	0.021	0.021	0.021	0.021	0.021	0.021	0.021	0.021	0.021	0.021	0.021	0.021	0.021	0.021	0.023	0.023	0.023	0.023	0.023	0.023	0.023	0.023	0.023	0.022	0.022	0.022	0.022	0.022	0.022	0.021	0.021	0.021	0.006	0.006	0.005	0.000	0.006	
	D7-5		0.022	0.022	0.022	0.022	0.022	0.022	0.022	0.022	0.022	0.022	0.022	0.022	0.022	0.022	0.022	0.022	0.022	0.024	0.024	0.024	0.024	0.024	0.024	0.024	0.024	0.024	0.023	0.023	0.023	0.023	0.023	0.023	0.022	0.022	0.022	0.006	0.006	0.006	0.006	0.000	

**Table A3:** Total Relation Matrix.

***T***=			D1-1	D1-2	D1-3	D1-4	D1-5	D1-6	D2-1	D2-2	D2-3	D2-4	D2-5	D3-1	D3-2	D3-3	D3-4	D3-5	D3-6	D4-1	D4-2	D4-3	D4-4	D4-5	D4-6	D4-7	D4-8	D4-9	D5-1	D5-2	D5-3	D5-4	D5-5	D5-6	D6-1	D6-2	D6-3	D7-1	D7-2	D7-3	D7-4	D7-5	
	D1-1		0.123	0.129	0.129	0.129	0.129	0.129	0.159	0.159	0.159	0.159	0.159	0.153	0.153	0.153	0.153	0.153	0.153	0.156	0.156	0.156	0.156	0.156	0.157	0.156	0.156	0.156	0.164	0.164	0.164	0.164	0.164	0.164	0.166	0.166	0.166	0.167	0.167	0.167	0.167	0.167	
	D1-2		0.122	0.117	0.122	0.122	0.123	0.122	0.150	0.150	0.150	0.150	0.150	0.145	0.145	0.145	0.145	0.145	0.145	0.148	0.148	0.148	0.148	0.148	0.148	0.148	0.148	0.148	0.155	0.155	0.156	0.155	0.155	0.156	0.158	0.158	0.158	0.158	0.159	0.158	0.158	0.158	
	D1-3		0.122	0.123	0.117	0.123	0.123	0.123	0.151	0.151	0.151	0.151	0.151	0.146	0.146	0.146	0.146	0.146	0.146	0.149	0.149	0.149	0.149	0.149	0.149	0.149	0.149	0.149	0.156	0.156	0.156	0.156	0.156	0.156	0.158	0.158	0.158	0.159	0.159	0.159	0.159	0.159	
	D1-4		0.127	0.128	0.127	0.121	0.127	0.127	0.157	0.157	0.157	0.157	0.157	0.151	0.152	0.152	0.152	0.151	0.151	0.154	0.155	0.154	0.154	0.154	0.155	0.154	0.154	0.154	0.162	0.162	0.162	0.162	0.162	0.162	0.164	0.164	0.164	0.165	0.165	0.165	0.165	0.165	
	D1-5		0.116	0.116	0.116	0.115	0.111	0.116	0.143	0.143	0.143	0.143	0.143	0.138	0.138	0.138	0.138	0.138	0.138	0.141	0.141	0.141	0.141	0.141	0.141	0.141	0.141	0.141	0.147	0.148	0.148	0.148	0.148	0.148	0.150	0.150	0.150	0.150	0.151	0.150	0.150	0.150	
	D1-6		0.112	0.112	0.112	0.112	0.113	0.107	0.138	0.138	0.138	0.138	0.138	0.133	0.134	0.134	0.134	0.133	0.134	0.136	0.136	0.136	0.136	0.136	0.137	0.136	0.136	0.136	0.143	0.143	0.143	0.143	0.143	0.143	0.145	0.145	0.145	0.146	0.146	0.146	0.145	0.146	
	D2-1		0.138	0.138	0.138	0.138	0.138	0.138	0.122	0.127	0.127	0.127	0.127	0.141	0.141	0.141	0.141	0.141	0.141	0.145	0.145	0.145	0.145	0.144	0.145	0.144	0.145	0.145	0.153	0.153	0.153	0.153	0.153	0.153	0.155	0.155	0.155	0.156	0.156	0.156	0.156	0.156	
	D2-2		0.133	0.134	0.134	0.133	0.134	0.134	0.123	0.118	0.123	0.123	0.124	0.136	0.137	0.137	0.137	0.137	0.137	0.140	0.140	0.140	0.140	0.140	0.140	0.140	0.140	0.140	0.148	0.148	0.148	0.148	0.148	0.149	0.150	0.150	0.150	0.151	0.151	0.151	0.151	0.151	
	D2-3		0.130	0.130	0.130	0.130	0.130	0.130	0.120	0.120	0.115	0.120	0.120	0.133	0.133	0.133	0.133	0.133	0.133	0.136	0.136	0.136	0.136	0.136	0.136	0.136	0.136	0.136	0.144	0.144	0.144	0.144	0.144	0.144	0.146	0.146	0.146	0.147	0.147	0.147	0.147	0.147	
	D2-4		0.130	0.130	0.130	0.130	0.130	0.130	0.120	0.119	0.120	0.115	0.120	0.133	0.133	0.133	0.133	0.133	0.133	0.136	0.136	0.136	0.136	0.136	0.136	0.136	0.136	0.136	0.144	0.144	0.144	0.144	0.144	0.144	0.146	0.146	0.146	0.147	0.147	0.147	0.147	0.147	
	D2-5		0.150	0.150	0.150	0.150	0.150	0.150	0.139	0.139	0.139	0.139	0.133	0.153	0.154	0.154	0.154	0.154	0.154	0.157	0.158	0.157	0.157	0.157	0.158	0.157	0.157	0.157	0.167	0.167	0.167	0.167	0.167	0.167	0.169	0.169	0.169	0.170	0.170	0.170	0.170	0.170	
	D3-1		0.139	0.139	0.139	0.139	0.139	0.139	0.148	0.148	0.148	0.148	0.148	0.119	0.126	0.126	0.126	0.125	0.125	0.147	0.147	0.147	0.147	0.147	0.147	0.147	0.147	0.147	0.154	0.154	0.154	0.154	0.154	0.155	0.156	0.156	0.156	0.155	0.155	0.155	0.155	0.155	
	D3-2		0.131	0.132	0.131	0.131	0.132	0.132	0.140	0.140	0.140	0.140	0.140	0.118	0.113	0.119	0.119	0.118	0.119	0.139	0.139	0.139	0.139	0.139	0.140	0.139	0.139	0.139	0.146	0.146	0.146	0.146	0.146	0.146	0.148	0.148	0.148	0.147	0.147	0.147	0.146	0.147	
	D3-3		0.129	0.129	0.129	0.129	0.129	0.129	0.138	0.137	0.137	0.137	0.138	0.116	0.117	0.111	0.117	0.116	0.116	0.137	0.137	0.137	0.137	0.137	0.137	0.137	0.137	0.137	0.143	0.144	0.144	0.144	0.144	0.144	0.145	0.145	0.145	0.144	0.144	0.144	0.144	0.144	
	D3-4		0.133	0.133	0.133	0.133	0.133	0.133	0.141	0.141	0.141	0.141	0.141	0.119	0.120	0.120	0.114	0.119	0.120	0.141	0.141	0.140	0.140	0.140	0.141	0.140	0.140	0.141	0.147	0.147	0.147	0.147	0.147	0.148	0.149	0.149	0.149	0.148	0.148	0.148	0.148	0.148	
	D3-5		0.126	0.126	0.126	0.126	0.126	0.126	0.134	0.134	0.134	0.134	0.134	0.113	0.114	0.113	0.113	0.108	0.113	0.133	0.133	0.133	0.133	0.133	0.133	0.133	0.133	0.133	0.140	0.140	0.140	0.140	0.140	0.140	0.141	0.141	0.141	0.140	0.141	0.140	0.140	0.140	
	D3-6		0.128	0.129	0.129	0.128	0.129	0.129	0.137	0.137	0.137	0.137	0.137	0.115	0.116	0.116	0.116	0.115	0.111	0.136	0.136	0.136	0.136	0.136	0.136	0.136	0.136	0.136	0.143	0.143	0.143	0.143	0.143	0.143	0.145	0.144	0.144	0.144	0.144	0.143	0.143	0.144	
	D4-1		0.122	0.122	0.122	0.122	0.122	0.122	0.130	0.130	0.130	0.130	0.130	0.126	0.127	0.127	0.127	0.126	0.127	0.109	0.116	0.115	0.115	0.115	0.116	0.115	0.115	0.115	0.139	0.139	0.139	0.139	0.139	0.139	0.142	0.142	0.142	0.142	0.142	0.142	0.142	0.142	
	D4-2		0.118	0.119	0.119	0.118	0.119	0.119	0.126	0.126	0.126	0.126	0.126	0.123	0.123	0.123	0.123	0.123	0.123	0.112	0.106	0.112	0.112	0.112	0.112	0.111	0.112	0.112	0.134	0.135	0.135	0.135	0.135	0.135	0.138	0.138	0.138	0.138	0.138	0.138	0.137	0.138	
	D4-3		0.117	0.117	0.117	0.117	0.117	0.117	0.124	0.124	0.124	0.124	0.124	0.121	0.122	0.122	0.122	0.121	0.121	0.111	0.111	0.105	0.111	0.110	0.111	0.110	0.110	0.111	0.133	0.133	0.133	0.133	0.133	0.133	0.136	0.136	0.136	0.136	0.136	0.136	0.136	0.136	
	D4-4		0.113	0.113	0.113	0.113	0.113	0.113	0.120	0.120	0.120	0.120	0.120	0.117	0.118	0.117	0.118	0.117	0.117	0.107	0.107	0.107	0.101	0.107	0.107	0.107	0.107	0.107	0.129	0.129	0.129	0.129	0.129	0.129	0.132	0.132	0.131	0.132	0.132	0.131	0.131	0.132	
	D4-5		0.114	0.114	0.114	0.114	0.114	0.114	0.121	0.121	0.121	0.121	0.121	0.118	0.118	0.118	0.118	0.118	0.118	0.107	0.108	0.107	0.107	0.102	0.108	0.107	0.108	0.108	0.129	0.129	0.129	0.129	0.129	0.130	0.132	0.132	0.132	0.132	0.132	0.132	0.132	0.132	
	D4-6		0.116	0.117	0.117	0.116	0.117	0.117	0.124	0.124	0.124	0.124	0.124	0.121	0.121	0.121	0.121	0.121	0.121	0.110	0.110	0.110	0.110	0.110	0.104	0.110	0.110	0.110	0.132	0.132	0.132	0.132	0.132	0.133	0.135	0.135	0.135	0.135	0.136	0.135	0.135	0.135	
	D4-7		0.114	0.114	0.114	0.114	0.114	0.114	0.121	0.121	0.121	0.121	0.121	0.118	0.118	0.118	0.118	0.118	0.118	0.107	0.107	0.107	0.107	0.107	0.108	0.102	0.107	0.107	0.129	0.129	0.129	0.129	0.129	0.129	0.132	0.132	0.132	0.132	0.132	0.132	0.132	0.132	
	D4-8		0.114	0.114	0.114	0.114	0.114	0.114	0.121	0.121	0.121	0.121	0.121	0.118	0.118	0.118	0.118	0.118	0.118	0.107	0.107	0.107	0.107	0.107	0.108	0.107	0.102	0.107	0.129	0.129	0.129	0.129	0.129	0.129	0.132	0.132	0.132	0.132	0.132	0.132	0.132	0.132	
	D4-9		0.112	0.112	0.112	0.112	0.112	0.112	0.119	0.119	0.119	0.119	0.119	0.116	0.117	0.116	0.117	0.116	0.116	0.106	0.106	0.106	0.106	0.106	0.106	0.106	0.106	0.100	0.127	0.128	0.128	0.128	0.128	0.128	0.130	0.130	0.130	0.131	0.131	0.130	0.130	0.131	
	D5-1		0.123	0.123	0.123	0.123	0.123	0.123	0.131	0.131	0.131	0.131	0.131	0.129	0.129	0.129	0.129	0.129	0.129	0.135	0.135	0.135	0.135	0.135	0.135	0.135	0.135	0.135	0.118	0.124	0.124	0.124	0.124	0.124	0.145	0.145	0.145	0.144	0.144	0.144	0.143	0.144	
	D5-2		0.119	0.120	0.120	0.119	0.120	0.120	0.127	0.127	0.127	0.127	0.127	0.125	0.126	0.126	0.126	0.125	0.125	0.131	0.131	0.131	0.131	0.131	0.131	0.131	0.131	0.131	0.120	0.115	0.121	0.120	0.120	0.120	0.141	0.141	0.141	0.140	0.140	0.140	0.139	0.140	
	D5-3		0.119	0.120	0.119	0.119	0.120	0.120	0.127	0.127	0.127	0.127	0.127	0.125	0.126	0.126	0.126	0.125	0.125	0.131	0.131	0.131	0.131	0.131	0.131	0.131	0.131	0.131	0.120	0.120	0.115	0.121	0.120	0.121	0.141	0.141	0.141	0.140	0.140	0.140	0.139	0.140	
	D5-4		0.110	0.110	0.110	0.110	0.110	0.110	0.117	0.117	0.117	0.117	0.117	0.115	0.116	0.116	0.116	0.115	0.116	0.121	0.121	0.121	0.121	0.121	0.121	0.121	0.121	0.121	0.110	0.111	0.111	0.105	0.111	0.111	0.130	0.130	0.130	0.129	0.129	0.128	0.128	0.129	
	D5-5		0.121	0.122	0.122	0.121	0.122	0.122	0.130	0.129	0.130	0.130	0.130	0.127	0.128	0.128	0.128	0.127	0.128	0.133	0.133	0.133	0.133	0.133	0.134	0.133	0.133	0.133	0.122	0.123	0.123	0.122	0.116	0.123	0.144	0.144	0.144	0.142	0.142	0.142	0.142	0.142	
	D5-6		0.110	0.110	0.110	0.110	0.110	0.110	0.117	0.117	0.117	0.117	0.117	0.115	0.116	0.116	0.116	0.115	0.116	0.121	0.121	0.121	0.121	0.121	0.121	0.121	0.121	0.121	0.110	0.111	0.111	0.111	0.111	0.106	0.130	0.130	0.130	0.129	0.129	0.128	0.128	0.129	
	D6-1		0.137	0.137	0.137	0.137	0.138	0.137	0.146	0.146	0.146	0.146	0.146	0.142	0.143	0.143	0.143	0.142	0.142	0.152	0.152	0.152	0.152	0.152	0.152	0.152	0.152	0.152	0.158	0.158	0.158	0.158	0.158	0.158	0.135	0.141	0.141	0.161	0.161	0.161	0.161	0.161	
	D6-2		0.135	0.135	0.135	0.135	0.135	0.135	0.144	0.143	0.143	0.143	0.144	0.140	0.140	0.140	0.140	0.140	0.140	0.149	0.149	0.149	0.149	0.149	0.149	0.149	0.149	0.149	0.155	0.155	0.155	0.155	0.155	0.156	0.139	0.133	0.139	0.158	0.158	0.158	0.158	0.158	
	D6-3		0.139	0.140	0.140	0.139	0.140	0.140	0.148	0.148	0.148	0.148	0.148	0.144	0.145	0.145	0.145	0.144	0.145	0.154	0.154	0.154	0.154	0.154	0.154	0.154	0.154	0.154	0.160	0.160	0.160	0.160	0.160	0.161	0.144	0.144	0.137	0.164	0.164	0.163	0.163	0.164	
	D7-1		0.114	0.114	0.114	0.114	0.115	0.114	0.121	0.121	0.121	0.121	0.121	0.118	0.119	0.119	0.119	0.118	0.119	0.123	0.123	0.123	0.123	0.123	0.123	0.123	0.123	0.123	0.128	0.128	0.128	0.128	0.128	0.128	0.130	0.130	0.130	0.110	0.116	0.115	0.115	0.116	
	D7-2		0.116	0.116	0.116	0.116	0.116	0.116	0.122	0.122	0.122	0.122	0.122	0.120	0.120	0.120	0.120	0.120	0.120	0.124	0.124	0.124	0.124	0.124	0.124	0.124	0.124	0.124	0.129	0.129	0.129	0.129	0.129	0.129	0.132	0.132	0.131	0.117	0.112	0.117	0.116	0.118	
	D7-3		0.119	0.119	0.119	0.119	0.119	0.119	0.126	0.126	0.126	0.126	0.126	0.123	0.124	0.124	0.124	0.123	0.124	0.128	0.128	0.128	0.128	0.128	0.128	0.128	0.128	0.128	0.133	0.133	0.133	0.133	0.133	0.133	0.136	0.136	0.136	0.121	0.121	0.115	0.120	0.121	
	D7-4		0.117	0.118	0.117	0.117	0.118	0.118	0.124	0.124	0.124	0.124	0.124	0.121	0.122	0.122	0.122	0.122	0.122	0.126	0.126	0.126	0.126	0.126	0.126	0.126	0.126	0.126	0.131	0.131	0.131	0.131	0.131	0.131	0.134	0.134	0.134	0.119	0.119	0.118	0.113	0.119	
	D7-5		0.122	0.122	0.122	0.122	0.122	0.122	0.129	0.129	0.129	0.129	0.129	0.126	0.127	0.127	0.127	0.127	0.127	0.131	0.131	0.131	0.131	0.131	0.132	0.131	0.131	0.131	0.137	0.137	0.137	0.137	0.137	0.137	0.139	0.139	0.139	0.124	0.124	0.124	0.123	0.118	

## Appendix D. Results of Sensitivity Analyses

**Table A4:** Sensitivity Analysis of the Ranking Results by VIKOR.

Exp.	Var.	Var. Value	VIKOR Values			Ranking			Exp.	Var.	Var. Value	VIKOR Values			Ranking		
			S	W	B	S	W	B				S	W	B	S	W	B
1	w_D1-1_	1.000	0.000	0.334	1.000	1	2	3	4	w_D1_	1.000	0.000	0.559	1.000	1	2	3
	w_D1-2_	1.000	0.000	0.715	1.000	1	2	3		w_D2_	1.000	0.000	0.779	1.000	1	2	3
	w_D1-3_	1.000	0.000	0.600	1.000	1	2	3		w_D3_	1.000	0.000	0.809	1.000	1	2	3
	w_D1-4_	1.000	0.000	0.222	1.000	1	2	3		w_D4_	1.000	0.000	0.750	1.000	1	2	3
	w_D1-5_	1.000	0.000	0.401	1.000	1	2	3		w_D5_	1.000	0.000	0.800	1.000	1	2	3
	w_D1-6_	1.000	0.000	0.143	1.000	1	2	3		w_D6_	1.000	0.000	0.634	1.000	1	2	3
	w_D2-1_	1.000	0.000	0.556	1.000	1	2	3		w_D7_	1.000	0.000	0.589	1.000	1	2	3
	w_D2-2_	1.000	0.000	1.000	0.857	1	3	2		w_D8_	1.000	0.000	0.491	1.000	1	2	3
	w_D2-3_	1.000	0.000	0.834	1.000	1	2	3	5	ν	0.000	0.000	0.331	1.000	1	2	3
	w_D2-4_	1.000	0.000	0.375	1.000	1	2	3			0.000	0.000	0.340	1.000	1	2	3
	w_D2-5_	1.000	0.126	0.000	1.000	2	1	3			0.000	0.000	0.350	1.000	1	2	3
	w_D3-1_	1.000	0.000	1.000	1.000	1	2	2			0.000	0.000	0.359	1.000	1	2	3
	w_D3-2_	1.000	0.000	0.500	1.000	1	2	3			0.000	0.000	0.369	1.000	1	2	3
	w_D3-3_	1.000	0.000	0.143	1.000	1	2	3			0.000	0.000	0.379	1.000	1	2	3
	w_D3-4_	1.000	0.000	1.000	1.000	1	2	2			0.000	0.000	0.388	1.000	1	2	3
	w_D3-5_	1.000	0.000	0.287	1.000	1	2	3			0.000	0.000	0.398	1.000	1	2	3
	w_D3-6_	1.000	0.000	0.778	1.000	1	2	3			0.000	0.000	0.407	1.000	1	2	3
	w_D4-1_	1.000	0.000	0.000	1.000	1	1	3			0.000	0.000	0.417	1.000	1	2	3
	w_D4-2_	1.000	0.250	0.000	1.000	2	1	3			0.000	0.000	0.427	1.000	1	2	3
	w_D4-3_	1.000	0.000	0.417	1.000	1	2	3									
	w_D4-4_	1.000	0.000	0.200	1.000	1	2	3									
	w_D4-5_	1.000	0.000	0.545	1.000	1	2	3									
	w_D4-6_	1.000	0.000	1.000	0.800	1	3	2									
	w_D4-7_	1.000	0.000	0.727	1.000	1	2	3									
	w_D4-8_	1.000	0.000	1.000	1.000	1	2	2									
	w_D4-9_	1.000	0.000	0.637	1.000	1	2	3									
	w_D5-1_	1.000	0.000	1.000	1.000	1	2	2									
	w_D5-2_	1.000	0.000	0.600	1.000	1	2	3									
	w_D5-3_	1.000	0.000	0.333	1.000	1	2	3									
	w_D5-4_	1.000	0.000	1.000	1.000	1	2	2									
	w_D5-5_	1.000	0.000	0.000	1.000	1	1	2									
	w_D5-6_	1.000	0.000	0.667	1.000	1	2	3									
	w_D6-1_	1.000	0.000	0.834	1.000	1	2	3									
	w_D6-2_	1.000	0.200	0.000	1.000	2	1	3									
	w_D6-3_	1.000	0.000	0.700	1.000	1	2	3									
	w_D7-1_	1.000	0.000	0.455	1.000	1	2	3									
	w_D7-2_	1.000	0.000	0.667	1.000	1	2	3									
	w_D7-3_	1.000	0.000	0.556	1.000	1	2	3									
	w_D7-4_	1.000	0.000	0.376	1.000	1	2	3									
	w_D7-5_	1.000	0.000	0.500	1.000	1	2	3									
2	All	Equal	0.000	0.762	1.000	1	2	3									
3	All	Rand.	Results of 100 Tests			1	2	3									

exp.—experience; var.—varaible; rand.—random.
